# The clinical features of polymerase proof-reading associated polyposis (PPAP) and recommendations for patient management

**DOI:** 10.1007/s10689-021-00256-y

**Published:** 2021-05-05

**Authors:** Claire Palles, Lynn Martin, Enric Domingo, Laura Chegwidden, Josh McGuire, Vicky Cuthill, Ellen Heitzer, The CORGI Consortium, Rachel Kerr, David Kerr, Stephen Kearsey, Susan K. Clark, Ian Tomlinson, Andrew Latchford

**Affiliations:** 1 Gastrointestinal Cancer Genetics Laboratory, Institute of Cancer and Genomic Sciences, College of Medical and Dental Sciences, University of Birmingham, Birmingham, B15 2TT UK; 2 Edinburgh Cancer Research Centre, Institute of Genetics and Cancer, University of Edinburgh, Crewe Road South, Edinburgh, EH4 2XU UK; 3 Department of Oncology, Old Road Campus Research Building, University of Oxford, Roosevelt Drive, Oxford, UK; 4 Wellcome Centre for Human Genetics, University of Oxford, Roosevelt Drive, Oxford, OX3 7BN UK; 5 Polyposis Registry, St Mark’s Hospital, Harrow, London, HA1 3UJ UK; 6 Diagnostic and Research Institute of Human Genetics, University of Gratz, Graz, Austria; 7 Nuffield Department of Clinical Laboratory Sciences, University of Oxford, John Radcliffe Hospital, Oxford, OX3 9DU UK; 8 ZRAB, University of Oxford, 11a Mansfield Road, Oxford, OX1 3SZ UK; 9 Department of Surgery and Cancer, Imperial College London, London, UK

**Keywords:** POLE, POLD1, PPAP, Exonuclease domain mutation

## Abstract

Pathogenic germline exonuclease domain (ED) variants of *POLE* and *POLD1* cause the Mendelian dominant condition polymerase proof-reading associated polyposis (PPAP). We aimed to describe the clinical features of all PPAP patients with probably pathogenic variants. We identified patients with a variants mapping to the EDs of *POLE* or *POLD1* from cancer genetics clinics, a colorectal cancer (CRC) clinical trial, and systematic review of the literature. We used multiple evidence sources to separate ED variants into those with strong evidence of pathogenicity and those of uncertain importance. We performed quantitative analysis of the risk of CRC, colorectal adenomas, endometrial cancer or any cancer in the former group. 132 individuals carried a probably pathogenic ED variant (105 *POLE*, 27 *POLD1*). The earliest malignancy was colorectal cancer at 14. The most common tumour types were colorectal, followed by endometrial in *POLD1* heterozygotes and duodenal in *POLE* heterozygotes. *POLD1-*mutant cases were at a significantly higher risk of endometrial cancer than *POLE* heterozygotes. Five individuals with a *POLE* pathogenic variant, but none with a *POLD1* pathogenic variant, developed ovarian cancer. Nine patients with *POLE* pathogenic variants and one with a *POLD1* pathogenic variant developed brain tumours. Our data provide important evidence for PPAP management. Colonoscopic surveillance is recommended from age 14 and upper-gastrointestinal surveillance from age 25. The management of other tumour risks remains uncertain, but surveillance should be considered. In the absence of strong genotype–phenotype associations, these recommendations should apply to all PPAP patients.

**Supplementary Information:**

The online version contains supplementary material available at 10.1007/s10689-021-00256-y.

## Introduction

It has historically been thought that Mendelian cancer predisposition syndromes are rarely, if ever, associated with a general predisposition to cancer. This is often perplexing, especially when the genes involved are ubiquitously expressed and/or are involved in fundamental processes such as cell metabolism or DNA repair. In fact, it is increasingly recognised that many of these syndromes confer a smaller, but definitely increased, risk of a wide range of cancers, this having been masked by a combination of early death from specific malignancies and ascertainment bias. Early reports of the spectrum of cancers associated with particular high-penetrance gene defects have often been revised in the light of improved treatment, longer follow-up and identification of gene heterozygotes in extended pedigrees. Almost without exception, these studies have confirmed that the cancers originally described are indeed the major risks, but lesser increased risks of other cancers are also present. Well established examples of the latter include prostate cancer in *BRCA1* and *BRCA2* heterozygotes [1], gastric cancer in individuals with Li-Fraumeni syndrome [2] and head and neck squamous cell cancer, gynaecological squamous cell cancer, oesophageal cancer, and liver, brain, skin and renal tumours in Fanconi anaemia patients [3].

It is therefore premature to report the clinical features of new Mendelian syndromes until several years have elapsed since their identification. This problem is compounded by the fact that the recently identified syndromes inevitably tend to be rarer, so that accumulation of clinical information is slow and it is almost impossible to avoid all ascertainment bias. A recent example of the confusion that can arise is the phenotype of the recessive condition *NTHL1-*associated polyposis (NAP), which initially was based on three families and largely comprises colorectal adenomas and carcinomas [4]; yet, a single NAP patient with seven primary cancers (and multiple non-cancerous tumours) has also been described [5]. Recent papers highlighting the now apparent extended tumour phenotype seen in individuals’ with two *NTHL1* mutations suggest *NTHL1* tumour syndrome as a more accurate name for this condition [6].

In this manuscript, we report the clinical features of a set of families with polymerase proofreading-associated polyposis (PPAP), a Mendelian dominant condition caused by pathogenic variants in the exonuclease domains of *POLE* and *POLD1*, the genes encoding the catalytic subunits of DNA polymerases epsilon and delta. We assess the phenotype of those heterozygous for an exonuclease domain (ED) probably pathogenic variant in the context of dysfunction of the proof-reading capability of these genes being expected to cause a 100-fold increase in point mutations in theoretically every dividing cell type in the body. We provide an update of the phenotype of the families when we first described PPAP [7] and combine this with a comprehensive literature review, resulting in a set of guidelines for PPAP management.

## Methods

### Patients

Eligible patients were identified from in-house studies (CORGI [8] – 2349 probands screened in 2013 (includes cases from *National Study of Colorectal Cancer Genetics (NSCCG))*, 2311 patients screened as part of this study, 1 proband identified as carrier by clinical genetics departments or VICTOR/QUASAR2 [9] – number of patients screened in 2013 = 1560, number of additional QUASAR 2 patients screened as part of this study = 287) or a literature review. 48 relatives of probands were screened for the family variant. Most of the cases reported in CORGI and all of the cases in QUASAR 2, were discovered by directly testing for *POLE* NP_006222.2:p. (Leu424Val) and *POLD1* NP_001243778.1:p. (Ser478Asn). One of the new families we report was identified by panel sequencing conducted at the clinical genetics centre that recruited the patient. All individuals with a *POLE* or *POLD1* ED variant were included, together with their personal and family histories, including as many details of tumours, screening and other major diseases as were available. For further details see Supplementary Methods.

### Variant annotation

Germline ED variant HGVS descriptions were validated using VariantValidator [10] (Supplementary Table S1) and variants were annotated with conservation scores (PhyloP), in silico predictions of likely impact on protein function (SIFT, POLYPHEN, Grantham) and frequency from gnomAD. Protein sequences of the exonuclease domains of human POLE and POLD1 were aligned using COBALT [11], selecting the identity setting for visualisation and scoring (red indicating full and blue partial conservation). The variants and the exo motifs (regions critical for exonucleolytic catalysis highly conserved between the two enzymes) were mapped onto the alignment (Supplementary Figure S1). We also identified studies that had assessed the function of POLE and *POLD1* ED variants in model organisms (e.g. *Schizosaccharomyces pombe)* or in vitro assays. We required an ED variant to fulfil at least one of the following criteria for classification as probably pathogenic: variant co-segregating with disease status in more than four meioses, hypermutation observed in yeast when the equivalent amino acid was mutated; or evidence of impaired proof-reading from biochemical assays (Supplementary Figure S2). We also required that where more than one of these was available, the data were compatible and consistent. The remaining ED variants were classified as of unknown significance (Supplementary Information, Supplementary Table S2). Details of the assessments of specific variants are provided in the Supplementary Information. The characteristics of the probably pathogenic variants in terms of frequency in gnomAD, in silico scores and location relative to exo motifs and conserved residues between POLE and POLD1 were also compared to the variants of uncertain pathogenicity and the differences observed inform the recommendations made in the discussion.

### Statistical analysis

Cumulative incidence of CRC, endometrial cancer (EC), colorectal polyps/adenomas and any cancer in probably pathogenic exonuclease domain variant heterozygotes was explored using survival analysis packages survival and survminer in R. Diagnosis of one of the above phenotypes was classed as an event and time of diagnosis was used as the time of event. If no event occurred and current age was known, this information was used to define the follow-up period. If no event occurred, but current age was not known, the last age at any other follow-up (for example, last clinic appointment or last cancer diagnosis) was used for censoring. If age at event was not reported the individual was excluded from the analysis.

## Results

### Patient ascertainment

In 2013, we reported eight PPAP families with germline *POLE* NP_006222.2:p. (Leu424Val), two families with *POLD1* NP_001243778.1:p. (Ser478Asn) and one individual with *POLD1* NP_001243778.1:p. (Pro327Leu) [6]. The pathogenic status of these variants has been demonstrated statistically and/or functionally. In this study, we updated and extended the families’ pedigrees and clinical details (Supplementary Figure S1, Supplementary Tables S3 and S4). Additional clinical information, including identification of three new heterozygotes, was also obtained for the *POLE* NP_006222.2:p. (Asn363Lys) family reported by Rohlin et al. [12]. We identified four new independent, unrelated *POLE* Leu424Val heterozygotes, two from the CORGI study (Fig. 2 Families M and N, Supplementary Table S3) and two, diagnosed with CRC at ages 46 and 37, in the QUASAR 2 trial (Supplementary Table S3). A total of 36 *POLE* Leu424Val heterozygotes and 11 of *POLD1* Ser478Asn heterozygotes, from 12 and two families respectively, was identified in this way (Supplementary Tables S3 and S4).

Other groups have reported pathogenic germline *POLD1* variants NP_001243778.1:p. (Asp316Gly), NP_001243778.1:p. (Asp316His) and NP_001243778.1:p. (Leu474Pro) in patients with colonic polyposis or CRC [13]. These variants co-segregated with affection status in samples from the index case’s family members and there is evidence from yeast and biochemical assays that mutation of these residues leads to impaired proof-reading. In our genotyping or sequencing data from 2311 colorectal polyposis or CRC patients (pedigrees O and P respectively in Fig. 2, clinical features in Supplementary Table S3) we identified NP_001243778.1:p. (Asp316Asn) in one family (two heterozygous individuals) and Leu474Pro in another (two heterozygous individuals). In CORGI 1% of patients screened were found to carry *POLE* Leu424Val, *POLD1* Ser478Asn, *POLD1* Asp316Asn *or POLD1* Leu474Pro and in QUASAR 2/VICTOR clinical trials 0.1% of CRC patients screened carried *POLE* Leu424Val or *POLD1* Ser478Asn.

**Fig. 1:**
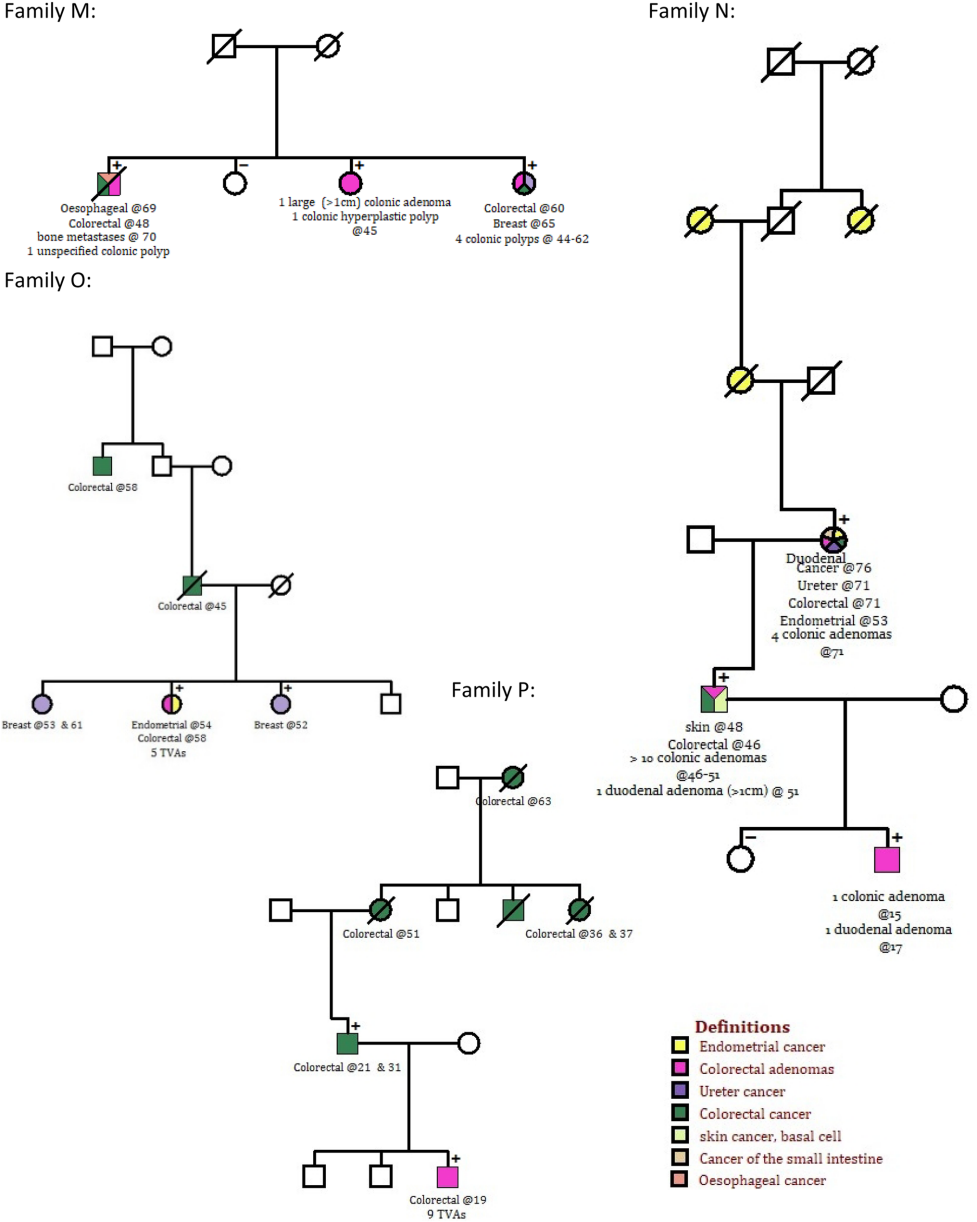
Pedigrees of the families in which new carriers of *POLE* and *POLD1* ED probably pathogenic variants were found

We performed a literature review to identify other individuals heterozygous for potentially pathogenic germline *POLE* and *POLD1* ED variants. Using criteria described in the Methods and Supplementary Information, we identified “probably pathogenic” *POLE* and *POLD1* germline ED variants – constitutional variants with at least one additional data type supporting their disease-causing effects – in 69 and 11 patients respectively (Fig. 1, Table 1, Supplementary Tables S3 and S4). Information on the 10 *POLE* and 9 *POLD1* variants identified from the literature, which did not meet our criteria for “probably pathogenic”, can be found in Supplementary Table S2.

Where available age at diagnosis and adenoma burden is indicated for each affected individual. + indicates an individual heterozygous genotype for the variant indicated, − indicates an individual who underwent genotyping but was negative.

**Table 1:** Summary of pathogenicity evidence and clinical manifestations for each germline variant in the exonuclease domains of *POLE* and *POLD1,* which has been reported in the literature and is presumed pathogenic

Protein change Transcript change	Evidence supporting pathogenicity of variant									Segregates with affection status∞	Number of heterozygotes (Number of unrelated heterozygotes)	Mean age at diagnosis (range)^b^	Number of heterozygotes with Cancer Type						Number of hetero-zygotes with adenomas		Other cancers reported in heterozygotes	References
	Structure	Conserved in POLD1-POLE alignment^c^	Grantham score	SIFT	Polyphen	Phylop score	Sig. greater# of mutations in yeastψ	Biochemical evidence for functional change^a^	GnomAD frequency				Colorectal	Endometrial	Breast	Duodenal	Ovarian	Brain	Duodenal	Colonic		
Polε																						
p.Thr278Lys c.833C > A	Exo 1 Motif	Partially	81	0	0.99	9.77	Yes [12]	Yes [13]	0	Yes	4 (1)	48 (39–54)	3	0	1	0	0	0	0	4		[12]
p.Asn363Lysc.1089C > A	ExoII Motif	Fully	94	0	1	− 0.66	NA	NR	0	Yes	23 (2)	42.6 (28–56)	17	2	0	3	3	5	1	8	Pancreas (N = 2), Prostate (N = 1)	[14, 15]
p.Asp368Val c.1103A > T	Exo II Motif	Fully	152	0	1	7.75	NR	Yes [16]	0	NR	1 (1)	47	1	0	0	0	0	0	0	0	None	[17]
p.Val411Leu c.1231G > C	Flanking Exo IV ( 8 bases away)	Partially	32	0	1	7.87	No [18]	Yes [19]	0	NA	1 (1)	14	1	0	0	0	0	0	1	1	None	[20]
p. Leu424Val c.1270C > G	ExoIV Moti	Fully	32	0	1	6.86	Yes [13, 18]	Yes[19]	0	Yes. 2 de novo heterozygotes	58 (22)	39 (16–64)	39	3	5	5	1	4	13	50	Basal cell carcinoma (N = 2), ureter N = 1), ampullary (N = 1). Urethra (N = 1), oesophagus (N = 1)	[7, 17, 21–24]
p. Tyr458Phe c.1373A > T	ExoIII motif	Fully	22	0	0.91	8.02	NA	Yes [25]	0	Yes	15 (2)	48 (28–63)	9	0	0	2 (1 jejun-um)	1		1	9	Pancreas (N = 1)	[26]
Polδ																						
p. Asp316Gly c.947A > G	Exo1 motif, active site	Fully	94	0.002	1	4.79	Yes [13]	Yes [16, 25]	0	Yes	2 (1)	51 (44–57)	1	2	1	0	0	0	0	1	None	[27]
p. Asp316His c.946G > C	Exo1 motif active site	Fully	81	0	1	7.03	Yes [13]	Yes [16, 25]	4.09 × 10^–6^	Yes	2 (1)	61 (58–64)	1	0	1	0	0	0	0	2	Mesothelioma (N = 1)	[27]
p. Asp316Asn c.946G > A	Exo1 motif active site	Fully	23	0.001	1	7.03	Yes [13]	Yes [16, 25]	0	Yes	2 (1)	53 (52–54)	0	1	1	0	0	0	0	1	None	NEW
p. Pro327Leu c.980C > T	Flanking ExoI motif (next base)	Fully	98	0	0.998	4.427	Yes [28]	Yes [19]^d^	0	NA	1(1)	70	0	0	0	0	0	0	0	1	None	[7]
p. Leu474Pro c.1421 T > C	ExoIV motif, paralogue of Leu424Val	Fully	98	0	1	3.04	Yes [13]	Yes [19]	0	Yes	8 (3)	35 (19–52)	5	2	0	0	0	0	0	2	None	[24, 27]
Ser478Asn c.1433G > A	ExoIV motif	Partially	46	0.002	0.996	5.207	Yes [7]	NA	0	Yes	12 (3)	36 (26–52)	5	4	1	0	0	1*	2	10	basal cell carcinoma (N = 1)	[7, 17]

Eligible patients were identified from in-house studies (CORGI [8] – 2349 probands screened in 2013 (includes cases from *National Study of Colorectal Cancer Genetics (NSCCG))*, 2311 patients screened as part of this study, 1 proband identified by clinical genetics departments OR VICTOR/QUASAR2 [9] – number of patients screened in 2013 = 1560, number of additional QUASAR 2 patients screened as part of this study = 287) or a literature review. 48 relatives of probands were screened for the family variant

A or P, adenomas or polyps; BrC, breast cancer; CRC, colorectal cancer; DuC, duodenal cancer; EC, endometrial cancer; GBM, glioblastoma; NR, not reported

*OC* ovarian cancer, *ODG* oligodendroglioma, *SIFT* Polyphen and PhyloP (100 way vertebrate) scores were obtained from dbNSFPv33a

^a^Data from functional studies of B family polymerases

^b^Mean age at diagnosis in years refers to cancer or adenoma diagnosis, whichever was earliest

^c^See methods, classification based up COBALT alignment[11]

^d^Functional studies of the corresponding residue in Pol ε

^#^Six heterozygotes were unaffected. **∞**

*Astrocytoma

POLE variants are annotated relative to transcript NM_006231.3 and protein accession NP_006222.2. POLD1 variants are annotated relative to transcript NM_001256849 and protein accession NP_001243778

### Tumour spectrum in PPAP

Individuals heterozygous for ED variants with probable or greater likelihood of being pathogenic (105 *POLE,* 27 *POLD1*) were included in the clinical phenotype assessment (Table 2, Supplementary Tables S3 and S4). Sex was available for 94 individuals.

**Table 2:** Summary information on polyp and cancer phenotypes observed in heterozygotes of likely pathogenic ED variants

Gene	Number of heterozygotes with specified cancer type (Median age at diagnosis)						Number of heterozygotes with adenomas (Median age at diagnosis)	
	Colorectal	Endometrial	Breast	Duodenal	Ovarian	Brain	Duodenal	Colonic
*POLE*	74/105 (44.5)	5/43 (53)	6/43 (49)	10/105 (54)	5/43 (45)	9/105 (35)	16/105 (51)	78/105 (36)
*POLD1*	12/27 (41)	9/17 (52)	4/17 (62)	0/17 (NA)	0/17 (NA)	1/27 (26)	2/27 (55)	18/27 (43)

Metastatic cancers were excluded from this summary

For endometrial, breast and ovarian cancers, counts in female heterozygotes are displayed

#### Colorectal tumours

One hundred (95%) *POLE* variant heterozygotes had a colorectal tumour phenotype (adenomas and/or carcinoma). The median ages at diagnosis of CRC and polyps were 44.5 and 36 respectively. Polyp burden was available for 59/78 *POLE* ED variant heterozygotes with polyps and ranged from one to 100, with a median of 12. Twenty-three of the 27 *POLD1* heterozygotes had a colorectal tumour phenotype. The median ages at diagnosis of CRC and polyps were 41 and 43 years respectively. Polyp burden was available for 17/18 heterozygotes with polyps and ranged from two to 70, with a median of 13.

Seventy of the 105 *POLE* variant heterozygotes developed CRC, at presentation or during follow-up (median age of diagnosis 43). 48 of these also had reported colorectal adenomas (CRAs). Age at diagnosis of CRAs was available for 40 of these patients; 10 patients had a history of adenoma removal prior to cancer diagnosis with the remained being diagnosed with adenomas synchronously with CRC or at a later time. Twelve of 27 (44%) *POLD1* variant heterozygotes developed CRC, none had prior adenoma removal and 5 had no reports of CRAs. The difference in frequency of CRCs and adenomas was not significantly different between *POLE* and *POLD1* heterozygotes (Fisher’s exact test, CRC p = 0.12, CRA p = 0.19).

Microsatellite instability (MSI) status was available for 29 colorectal tumours from *POLE* variant heterozygotes and three from *POLD1* variant heterozygotes; four (all carcinomas from *POLE* variant heterozygotes) of 28 CRCs (14.3%) and no adenomas were MSI + . Loss of mismatch repair (MMR) proteins (assessed by immunohistochemistry) was reported in CRCs from three of the four MSI cases. No information regarding MMR protein loss was available for the fourth case, just the number of microsatellites that were unstable.

Most colorectal polyps were tubular or tubulovillous adenomas, although nine *POLE* and five *POLD1* variant heterozygotes also had hyperplastic polyps (Supplementary Tables S3 and S4). Sixteen variant heterozygotes were diagnosed with multiple CRCs. Ten of these were synchronous cancers involving 2–4 tumours and in the remainder, CRC developed 1–22 years later (median 10 years). Those developing multiple CRCs carried *POLE* Leu424Val (N = 10), *POLE* Thr278Lys (N = 2), *POLE* Asn363Lys (N = 2), *POLD1* Ser478Asn (N = 1), POLD1 Leu474Pro (N = 1).

Information on surgery was available for 24 participants, of whom 19 underwent colectomy (4 segmental and 15 extended) following CRC diagnosis. Based on available information, in three cases colectomy (reported as one pan-proctocolectomy, one extended left hemi-colectomy and one right hemi-colectomy) was chosen following metachronic cancer and a further four participants underwent prophylactic surgery following diagnosis of multiple bowel adenomas (one proctocolectomy, one subtotal and one total colectomy; one underwent “colectomy” but further details of this surgery are not available).

All CORGI participants with probably pathogenic ED variant were undergoing colonoscopic surveillance; one developed cancer on surveillance and one developed cancer after having been discharged from follow-up.

#### Upper gastrointestinal tumours

After colorectal tumours, duodenal tumours were the next most frequent lesions in *POLE* variant heterozygotes. Ten of 105 (9.5%) probably pathogenic *POLE* ED variant heterozygotes developed duodenal cancer (DC; median age 55) and 16 (15%) developed one or more duodenal adenomas (DAs; median age 43). 23 probably pathogenic *POLE* ED variant heterozygotes developed both a colorectal phenotype (CRC and or CRAs) and a duodenal phenotype (DC and or DAs). In two cases, DAs arose before age 18. No DCs have arisen to date in *POLD1* ED heterozygotes, but DAs were diagnosed in 2/27 cases (9%; median age 55) who also had CRC and or CRAs. The difference in frequency of DC and DA was not significant (Fisher’s exact test DC p = 0.21, DA p = 0.36).

#### Endometrial and ovarian cancers

**Fig. 2:**
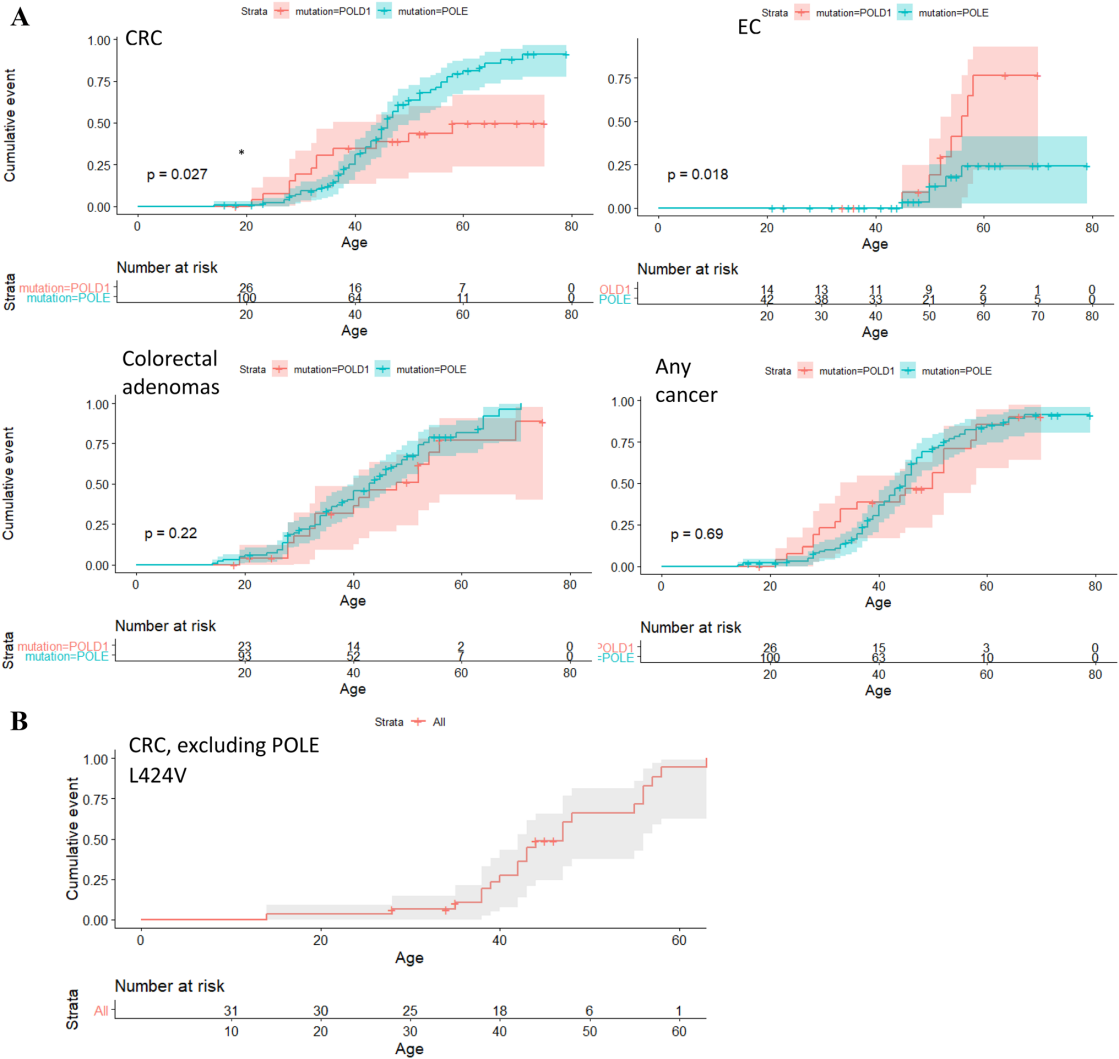
Graphs showing the cumulative risk of developing CRC, colorectal adenomas, endometrial cancer and any cancer in carriers of probably pathogenic variants in *POLE* and *POLD1*

EC was the most common malignancy in female *POLD1* variant heterozygotes (9/17, 53%), with three diagnosed under the age of 50. 5/43 *POLE* variant heterozygotes (12%) were diagnosed with EC, one before the age of 50. The frequency of EC was significantly lower in *POLE* variant heterozygotes (Fisher’s exact test, p = 0.001) (Fig. 2a). Five female *POLE* ED variant heterozygotes developed ovarian cancer (OC) between the ages of 33 and 45, including one with bilateral disease age 40 years, but no OCs were found in *POLD1* ED variant heterozygotes (Fisher’s exact test, p = 0.31)). Two patients were diagnosed with OC at the same time as their EC, although it was not clear whether the synchronous lesions had independent origins. No further details on the histology or molecular biology of the ovarian cancers were available. Data were also incomplete with respect to premalignant gynaecological lesions. However, two POLE Leu424Val heterozygotes underwent hysterectomy for benign pathology, specifically endometrial dysplasia or hyperplasia, or endometriotic cysts.

Out of a total of 105 POLE carriers and 27 POLD1 carriers, of which 43 POLE carriers and 17 POLD1 carriers were female, the following had sufficient information to be included in the analysis of cumulative risk of (1) CRC: POLE N = 103 POLD1 N = 27 (2) EC POLE N = 43, POLD1 N = 13, (3) colorectal adenomas POLE N = 87, POLD1 N = 25 (4) any cancer POLE N = 94 POLD1 N = 27. a Gene stratified analysis: Male and female heterozygotes were analysed together apart from in the EC plot which was generated using data from female heterozygotes only. “Any cancer” includes brain tumours but not duodenal or colorectal adenomas. No correction was made for screening or surgical interventions. b CRC cumulative risk in POLE presumed pathogenic variant heterozygotes, excluding POLE Leu424Val. Shaded areas represent 95% confidence intervals *P-value remains significant when POLE Leu424Val heterozygotes are excluded, p=0.018.

#### Brain tumours

Brain tumours (two astrocytomas at age 26) were observed in one of the first *POLD1* Ser478Asn heterozygotes described. Brain tumours have also now been reported in nine of 105 (9%) of *POLE* variant heterozygotes. Two *POLE* Leu424Val heterozygotes developed glioblastomas at age 47 and age 61 respectively, one Leu424Val heterozygote developed an astrocytoma at age 15 and one Leu424Val heterozygote developed an oligodendroglioma aged 30. Five Asn363Lys heterozygotes developed primary brain tumours, comprising four glioblastomas diagnosed between ages 30 and 52, and one of unspecified type at 35.

#### Other common tumours

Breast cancer was not described in the original PPAP families, but there are now multiple reports of breast cancer in female patients with PPAP. Six of 43 (14%) female *POLE* variant heterozygotes developed breast cancer (median age 49 years, range 38–65), as did 4/17 (24%) female *POLD1* variant heterozygotes (median age 62 years, range 52–65). The latter frequency is clearly higher than the general population. Despite some recent reports that *POLE* and *POLD1* variants predispose to prostate cancer [29], only one patient developed prostate cancer (aged 51) in the families analysed.

#### Multiple tumours

15/51 (29%) male and 20/43 (46.5%) female *POLE* ED variant heterozygotes developed benign or malignant tumours of more than one site to the date of follow-up. For *POLD1,* the equivalent frequencies were 2/10 (20%) males and 10/17 (59%) females. Three out of 132 patients had synchronous tumours of different organs.

### Penetrance of probably pathogenic variants

**Fig. 3:**
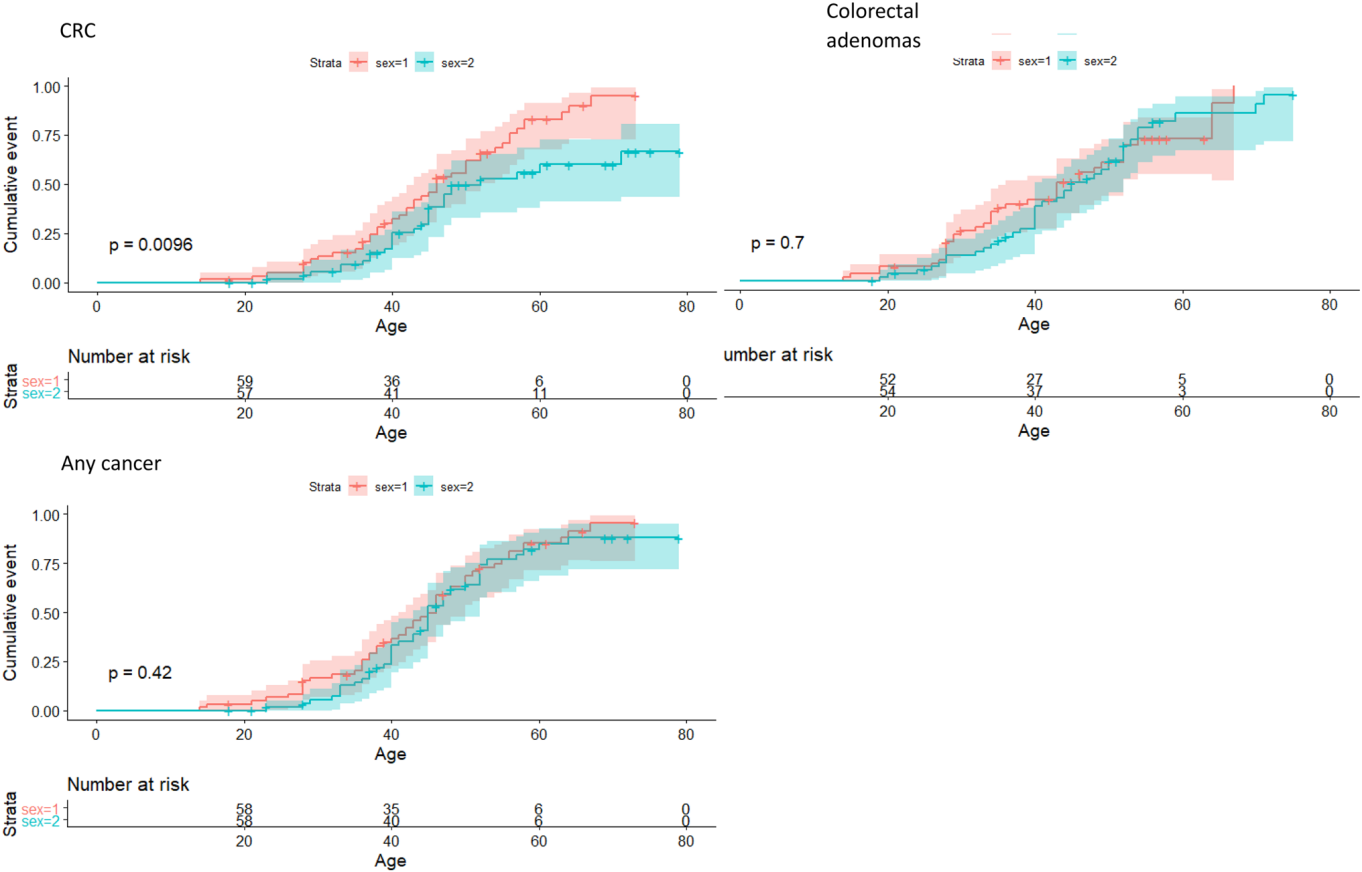
Graphs showing the cumulative risk of developing CRC, colorectal adenomas and any cancer in male and female carriers of probably pathogenic variants in *POLE* and *POLD1.* *POLD1* and *POLE* ED variant heterozygotes have been grouped together. Shaded areas represent 95% confidence intervals

Cumulative incidence curves (Fig. 2a) show the age-dependent penetrance of the following phenotypes in those heterozygous for a probably pathogenic *POLE* and *POLD1* ED variant: CRC; colorectal adenoma(s); EC; and any cancer including brain tumour. 15 patients developed CRC before age 30. The risk of CRC by age 70 years was approximately 90% for *POLE* variant heterozygotes and lower (around 50%) for *POLD1* ED variant heterozygotes (p = 0.027). Conversely, *POLD1* ED variant heterozygotes had a higher risk over time than *POLE* ED variant heterozygotes of developing EC (P = 0.018). The risk of EC by age 70 was in the region of 75% for *POLD1* and about 25% for *POLE* variant heterozygotes. No ECs developed before age 45 years in any of these variant heterozygotes. Males had a higher lifetime risk of CRC than females (P = 0.0096; Fig. 3) for both genes, although this association was not present for colorectal adenomas or when cancers of any organ were considered (Fig. 3). We compared CRC incidence between those heterozygous for the prototypic Leu424Val variant and other variants, but no clear difference was found (Fig. 2b).

## Discussion

In this study, we have provided a comprehensive description of the clinicopathological features of PPAP in those heterozygous for POLE and POLD1 ED variants with supporting evidence of a pathogenic impact. The cumulative incidence of CRC in *POLE* and *POLD1* variant heterozygotes is estimated at approximately 90% and 50% respectively. These estimates are higher than previous reports [30], probably because of the exclusion of variants of uncertain pathogenicity and the variable mixture between studies of PPAP patients who were screened because of family history and others whose first cancer occurred before surveillance began. Most patients have an attenuated polyposis phenotype (10–100 polyps), with greater polyp numbers in a few cases, but a minority of cases have a Lynch syndrome-like phenotype with early-onset CRC and few polyps. Individuals without CRAs were either young (aged 24 and 18 respectively), or carried a relatively rare probably pathogenic variant and presented with an extra-colonic tumour (*POLE* p.Tyr458Phe, *POLD1* p. Asp316Gly, *POLD1* p. Asp316Asn, *POLD1* p. Leu474Pro).

We have expanded the spectrum of intestinal cancers to include benign and malignant duodenal tumours. 19% of all probably pathogenic ED variant heterozygotes had both a colorectal and a duodenal phenotype. Unfortunately, phenotypic data regarding the upper GI tract are somewhat limited and indeed many of the patients reported in the literature are likely not to have undergone upper GI endoscopy at all. When only those who have had an upper GI endoscopy were included, DAs were seen in over 50% of ED variant heterozygotes.

EC is the most common extra-intestinal malignancy in PPAP, with lower risks than CRC. The risk is higher in *POLD1* heterozygotes. OC is also relatively frequent, sometimes synchronous with EC, although no consistent assessment of histology or clonal origins was possible. There is also growing evidence that a variety of brain tumours can occur as part of PPAP.

Caution is advised in interpreting the details of some of our findings, including the quantitative cancer risk estimates and the intriguing possibility of genotype–phenotype correlations. Potential problems include the small numbers of affected individuals from a limited number of families, the variable extent of cancer screening, and ascertainment bias for genetic testing. Such issues are unavoidable for rare conditions, and must be take into account wherever possible in the clinical setting.

How do our data inform clinical management? Colorectal surveillance is clearly advisable. Bellido et al. [27] suggested colonoscopy from the age of 18 years. Given that we identified an individual with a CRA aged 15 and Wimmer et al. [20] identified a CRC in a 14 year old, we suggest that earlier colonoscopic assessment at age 14 should be performed in all PPAP patients. It is noteworthy that 26/86 (30%) CRC cases were in patients who did not have adenomas and no clear correlation between polyp burden and cancer risk has been demonstrated. Furthermore, although the tumours we originally described in *POLE* and *POLD1* carriers were microsatellite-stable, the frequency of MSI (12.5%) that we now describe in CRCs is similar to that in sporadic CRCs, despite the earlier onset of the former. Whether MSI in PPAP CRC results from somatic loss of mismatch repair or an unappreciated direct effect of constitutive proofreading deficiency, it could result in accelerated progression to cancer. Given the above uncertainties, we recommend at least biennial colonoscopy, compared with 1–2 yearly suggested by Bellido et al. [27]. Prophylactic surgical intervention may be appropriate for PPAP cases with a severe polyp phenotype: our data suggest that colectomy and ileo-rectal or ileo-distal sigmoid anastomosis would usually be most appropriate. Surgery for those with PPAP in whom CRC has already arisen needs to be individualised and no strong recommendation can be given; factors including phenotype, expected functional outcome and the metachronous cancer risk all need to be considered.

The finding of frequent DAs and DC suggests that routine upper gastrointestinal tract surveillance would be beneficial. In the absence of robust data, we suggest that surveillance is based on the system advocated for FAP [31], starting at age 25. The role of duodenal polypectomy is unproven, even in FAP, but it would seem reasonable to consider polypectomy in PPAP when adenomas reach 1 cm in size.

Endometrial cancer is common in PPAP and it is logical to think that gynaecological surveillance would be warranted. Indeed, Bellido and colleagues [27] proposed endometrial surveillance in patients with *POLD1* pathogenic variants, commencing age 40 years. However, there are no robust data to support gynaecological screening in Lynch syndrome, which has a comparable or higher risk of gynaecological cancer, even though it is performed in some centres. Instead of gynaecological surveillance, Lynch syndrome patients are usually counselled regarding the possibility of risk-reducing surgery when they have completed their family [32]. For *POLE* carriers, given the finding of 5 cases with ovarian cancer, this counselling should include a discussion of both risk reducing hysterectomy and bilateral salpingo-oophorectomy (BSO). We suggest a similar approach for women with PPAP, since the EC risk is appreciable with either *POLD1* or *POLE* ED variant heterozygotes.

For the other cancers observed in PPAP families, the absolute risk appears relatively small and below the threshold at which routine surveillance would be recommended. The lifetime risk of breast cancer in the female UK population is 12% and the risk of developing breast cancer in the next 10 years if aged 50 is 3.54% whereas the risk of breast cancer by age 60 is approximately 30% in POLD1 and 20% in *POLE* ED pathogenic variant heterozygotes included in our analysis (Supplementary Figure S4). Whilst this is based on only 40 female *POLE* ED heterozygotes and 17 female *POLD1* heterozygotes, we conclude that female *POLD1* ED heterozygotes in particular may be at an increased risk of developing breast cancer compared to the general population, but this is not proven. Specifically, there is, as yet, insufficient evidence that the risk observed is high enough to fulfil the criteria for moderate risk breast screening according to UK NICE (Familial breast cancer: classification, care and managing breast cancer and related risks in people with a family history of breast cancer, Clinical guideline [CG164] updated: March 2017 [33]). We suggest *POLE* and *POLD1* ED heterozygotes could be referred to a high risk breast screening service for discussion. The lack of data about type of breast cancer, hormone receptor status and the inherent bias in our cohort make it difficult to give robust recommendations. Discussion in a specialised unit is sensible particularly where there is clustering of breast cancer cases or the presence of very young onset breast cancer in a first degree relative. Certainly, breast awareness and self-examination should be encouraged in all female patients with PPAP. Whilst there are no data to support that it is effective, increasing awareness and empowering patients to be involved in the management of their condition should be supported.

The PPAP phenotype is in some ways a hybrid between attenuated adenomatous polyposis (as seen in other DNA repair deficiency syndromes such as MAP) and Lynch syndrome, especially as regards the high EC risk. CRC and EC cancer patients with somatic *POLE* ED mutations have been shown to have a favourable prognosis [34, 35] and it would be interesting in future studies to investigate whether the prognosis of cancer patients with germline *POLE* or *POLD1* mutations is also better. There is currently no consensus as to who should have diagnostic testing for PPAP (screening of EDs of *POLE* and *POLD1* for pathogenic variants); we suggest that it should be considered for those with unexplained adenomatous polyposis, as well as those with a family history fulfilling the Amsterdam or modified Bethesda criteria, especially if no pathogenic mismatch repair variants have been found. Distinguishing pathogenic, PPAP-causing variants from non-pathogenic variants in *POLE* and *POLD1* may be challenging. Based on the features of the mutations described here and the likely absence of functional data to help assessment of novel mutations in the clinic, we suggest applying the following filtering steps to distinguish a likely pathogenic variant:

1. Maps to the exonuclease domain of POLE (amino acids 268–471) or POLD1 (amino acids 304–533)
2. Allele frequency of < 1 × 10^–5^ in non-Finnish European gnomAD data
3. Maps to or flanks an exo motif and affects an amino acid that is perfectly or highly conserved in a POLE-POLD1 protein alignment
4. Classed as pathogenic by two or more in silico tools
5. Results in a protein predicted to *retain* polymerase and regulatory functions.

Additional DNA samples from relatives for co-segregation studies, and from tumours for mutation burden, spectrum and signature analysis are also very helpful. It remains desirable to find an in vitro system for rapid testing of novel variants. Design of functional assays in accordance with American College of Medical Genetics and Genomics (ACMG) and the Association for Molecular Pathology (AMP) guidelines would aid greatly with variant interpretation [36]. The set of variants selected here as probably pathogenic all had supporting evidence from at least one of the following: co-segregation studies, mutator phenotype assessment in yeast assays or biochemical proof reading assays, in addition to fulfilling the five criteria above. If any of these supporting lines of evidence were conflicting or inconclusive, the variant was classed as being of unknown significance (e.g. *POLE* W347C and *POLE* L460M). It is possible that we have excluded variants that are pathogenic, but we decided that strict classification criteria for pathogenic variants was required in order to give an accurate survey of the clinical features of those with PPAP.

This manuscript is a step forward in describing the clinical features of PPAP based on variants with strong evidence of pathogenicity with implications for diagnostic testing algorithms and hence screening. With time, the understanding of this condition and its cancer risk will no doubt evolve and inform updates to our recommendations for clinical management. We highlight the difficulties in identifying truly pathogenic variants and provide suggestions that could be incorporated in a more formal variant classification system for suspected PPAP-causing variants.

## Supplementary Information

Below is the link to the electronic supplementary material.

Supplementary file1 (DOCX 1166 kb)

## Data Availability

Individual level information for all patients identified during this research can be found in the supplementary tables.
