# Supplementary material for: The clinical features of polymerase proof-reading associated polyposis (PPAP) and recommendations for patient management
Source: Fam Cancer. 2021 May 5;21(2):197–209. doi: 10.1007/s10689-021-00256-y (PMC8964588; doi:10.1007/s10689-021-00256-y)
Supplement: Supplementary file 1 — Supplementary file1 (DOCX 1166 kb) [file 10689_2021_256_MOESM1_ESM.docx]

**Supplementary Information**

The CORGI Consortium

Dr Kai Ren Ong, Birmingham Women's Hospital, Birmingham, Prof. Andrew Beggs, Institute of Cancer and Genomic Sciences, University of Birmingham, Dr. Alan Donaldson, St. Michael's Hospital, Dr. Ruth Armstrong, Addenbrooke's NHS Trust, Cambridge, Dr. Carole Brewer, Royal Devon & Exeter Hospital (Heavitree), Exeter, Prof. Jayantha Arnold, Ealing Hospital, Middlesex, Dr. Munaza Ahmed, Great Ormond Street Hospital, London, Dr. Louise Izatt, Guy's Hospital**,** London, Dr. Andrew Latchford, Northwick Park & St. Marks Hospital, Harrow, Prof. Dorothy Halliday, The Churchill Hospital, Oxford, Peter Risby, The Churchill Hospital, Oxford, Dr Paul Brennan, The James Cook University Hospital, Middlesbrough, Dr. Alison Kraus, Chapel Allerton Hospital, Leeds, Dr. Julian Barwell, Leicester Royal Infirmary, Leicester, Dr. Lynn Greenhalgh, Liverpool Women's Hospital, Prof. D. Gareth Evans, St Mary's Hospital, Manchester, Kate Green St Mary's Hospital, Manchester, Dr. Timothy Simmons, Institute of Genetic Medicine, International Centre for Life, Newcastle upon Tyne, Dr. Rachel Harrison, City Hospital Campus, Nottingham, Prof. Ragunath, Queen's Medical Centre Campus, Nottingham, Prof. Brian Davidson, Royal Free Hampstead NHS Trust, University Dept. of Liver Medicine & Transplantation, London, Dr. Zoe Kemp, The Royal Marsden, Sutton, Dr. Helen Hanson, St George’s University, London, Prof. Anneke Lucassen, Princess Anne Hospital, Southampton, Dr. Kevin J Monahan West Middlesex University Hospital, Middlesex, Professor Patrick Morrison, City Hospital Campus, Belfast.

**Supplementary Methods**

**Further details on the assessment of the pathogenicity of EDM mutations from the literature**

The features of the genetic variants classified as “probably pathogenic” and the phenotypes of carriers identified from CORGI, QUASAR 2 or the literature review are shown in Table 1. Of the 12 mutations deemed probably pathogenic, ten affected sites within Exo motifs I-IV, two flanked an Exo motif and none involved the ExoV motif. All of variants apart from POLE N363K had supporting evidence of a functional impact on proof reading ability (see Table 1). POLE N363K has been identified in two independent large families with co-segregation extending beyond four meioses. 82% of the presumed pathogenic variants mapped to a site that was fully conserved between POLE and POLD1 (Supplementary Figure 1).

Only 2 (11%) of the variants in Supplementary Table 1 with insufficient supporting evidence to be classed as pathogenic – and hence deemed of unclear functional significance – were fully conserved between the two proteins. The two variants at fully conserved sites were *POLE* p.W347C and *POLD1* p.R409W. *POLE* p.W347C was discovered in a family with a very different phenotype to that seen in most PPAP families, with a predominant phenotype of cutaneous melanoma and no colorectal phenotype. The lack of co-segregation of the variant with cutaneous melanoma and the lack of colorectal involvement resulted in it being classed as having unclear pathogenic status. *POLD1* p.R409W was reported in a single case with CRC and adenomas. No co-segregation data or analysis of impact on proof reading was available.

The *POLE* D287E mutation (Supplementary Table 1) warrants mention, as this is a recurrent germline variant that flanks the Exo I motif, has been reported to be pathogenic by several studies[1-3] and has been associated with multiple cancer types. It sits next to the most frequent site of somatic *POLE* mutation, codon 286, and is present in the ExAC database at a frequency of about 0.001. However, it has a low PhyloP conservation score and fails to co-segregate with disease (Supplementary Table 1). We performed functional assessments of the analogous mutation to D287E using fluctuation analysis in *S. pombe* and found no evidence of an increased mutation rate compared to wildtype yeast (Supplementary Figure 2). Furthermore, we screened 2,311 polyposis and CRC patients for *POLE* p.D287E and identified three heterozygous carriers. DNA from an affected family member was available for one carrier and the variant failed to co-segregate. The frequency of *POLE* p.D287E in CRC and polyposis cases was, moreover, in line with that observed in non-Finnish Europeans in gnomAD (minor allele frequency in CRC and polyposis cases 0.0013, minor allele frequency in gnomAD 0.0018). Overall, *POLE* p.D287E cannot be classified as probably pathogenic based on current evidence.

*POLE* V411L was retained in the set of likely pathogenic germline mutations. Barbari et al showed that this variant did not display a hypermutator phenotype in yeast. V411L also maps more than 1 base away from an Exo motif (Supplementary Figure 1) and is also only partially conserved with POLE. However, V411L has a 3-fold reduction in exonuclease activity compared to wild type[4] and, most importantly, is a highly recurrent somatic mutation in sporadic CRCs and ECs[5-7]. We therefore decided that the weight of evidence supported pathogenicity of this variant in the germ line.

The set of variants selected here as probably pathogenic all had supporting evidence from at least one of the following: co-segregation studies, mutator phenotype assessment in yeast assays or biochemical proof reading assays, in addition to fulfilling the five criteria above. If any of these supporting lines of evidence were conflicting or inconclusive, the variant was classed as being of unknown significance (e.g. *POLE* W347C and *POLE* L460M). It is possible that we have excluded variants that are pathogenic. We applied strict classification criteria for pathogenic variants in order to give an accurate survey of the clinical features of those with PPAP.

**Supplementary Figure 1:**

**Exonuclease domains of human *POLE* and *POLD1* with reported probably pathogenic variants and variants of unknown pathogenicity marked**


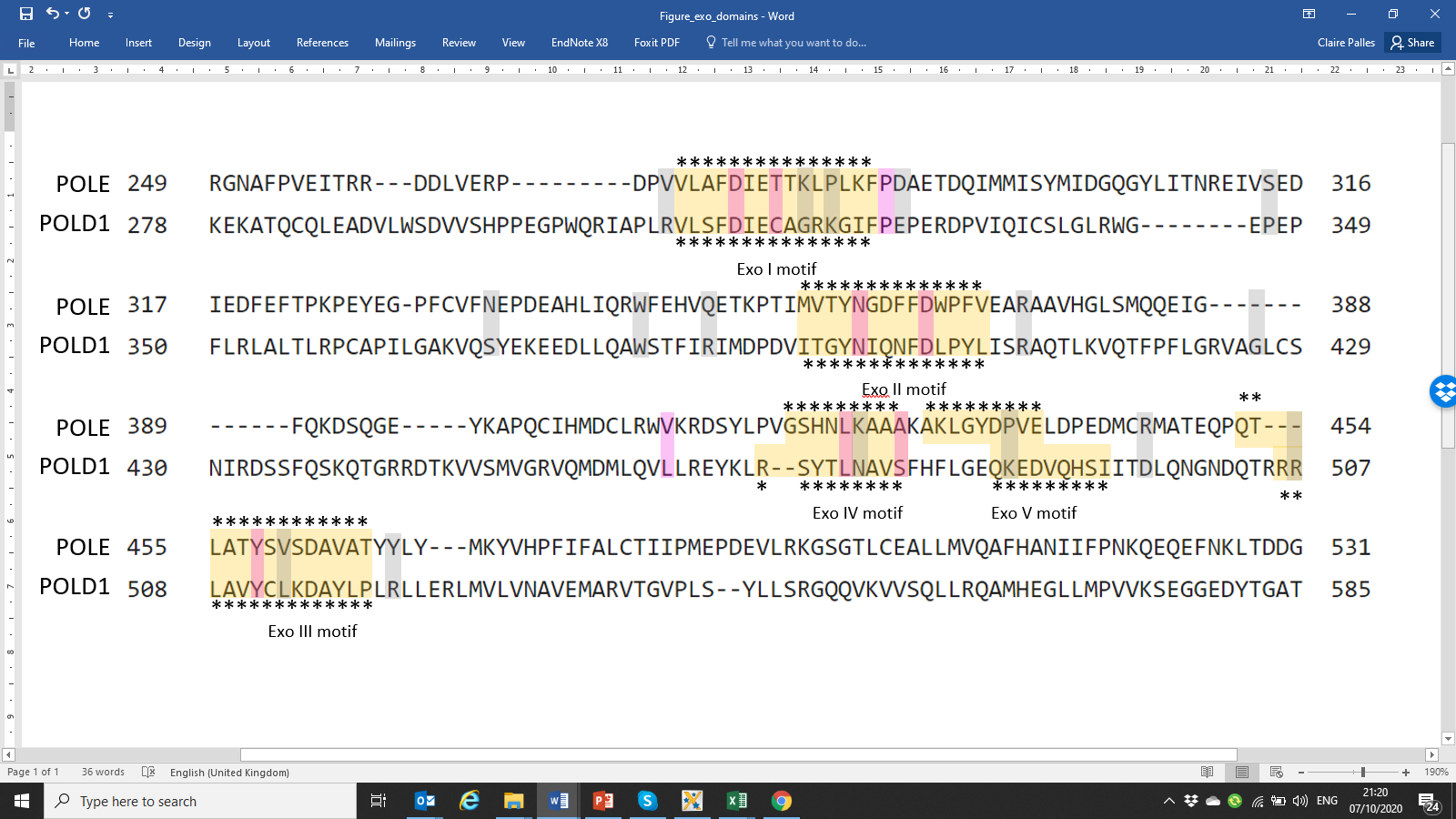


POLE and POLD1 protein sequences were aligned using COBALT. Exo domains are marked in yellow and with asterisks. Probably pathogenic variants are marked in purple with variants of unknown pathogenicity in grey.

**Supplementary Figure 2: Assessment of mutator phenotype of selected *POLE* variants in *S.pombe***

To construct yeast strains harbouring the *POLE* variants site directed mutagenesis was performed and the PCR products harbouring the mutation were cloned into pFA-kanMX6. Subsequentially, plasmids were linearized and integrated into *S.pombe* 2840. POLE WT is a strain constructed in the same way as the variants which involved a partial duplication of the pol e gene, we made it to check that the method of strain construction does not affect the mutation rate. To calculate the mutation rates, fluctuation analysis originally described by Luria and Delbruck (1943), was performed. After initial growth in in a non-selective medium, cells are plated onto selective media to obtain the number of mutants, r, and dilutions are plated onto rich medium to calculate the total number of viable cells. Three different assays were performed including ADE- to ADE+ (Stop codon has to be reverted), FOA-resistance (URA+ to URA-) (ura4 or ura5 have to be inactivated) and CAN-resistance (CAN+ to CAN-) At least 2 biological replicates were used (two independently generated strains). Mutation rate of the constructed strains were compared to the wild type (WT) using a T-test. “*” indicates significance at p<0.05 in two-sided T-test.

**Supplementary Figure 3: Updated Pedigrees of families with POLE L424V or POLD1 S478N mutations, first reported in 2013(Palles et al)**

**Family B**

**
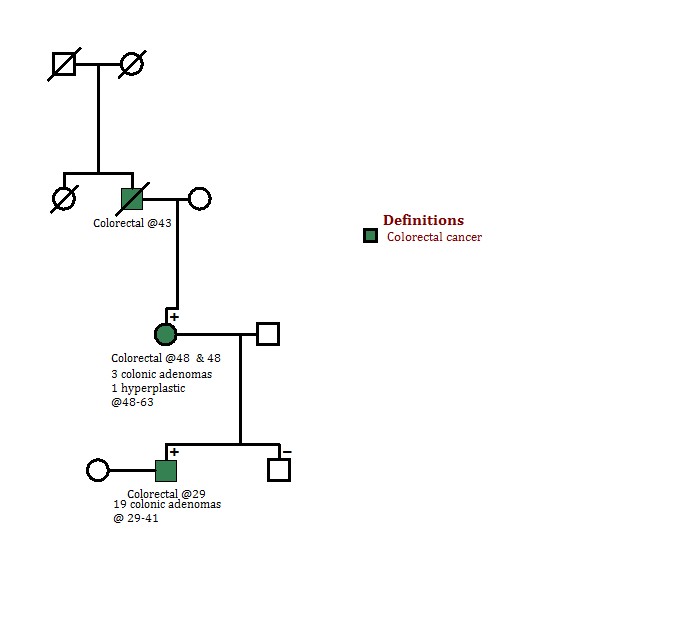
**

**Family C**

**
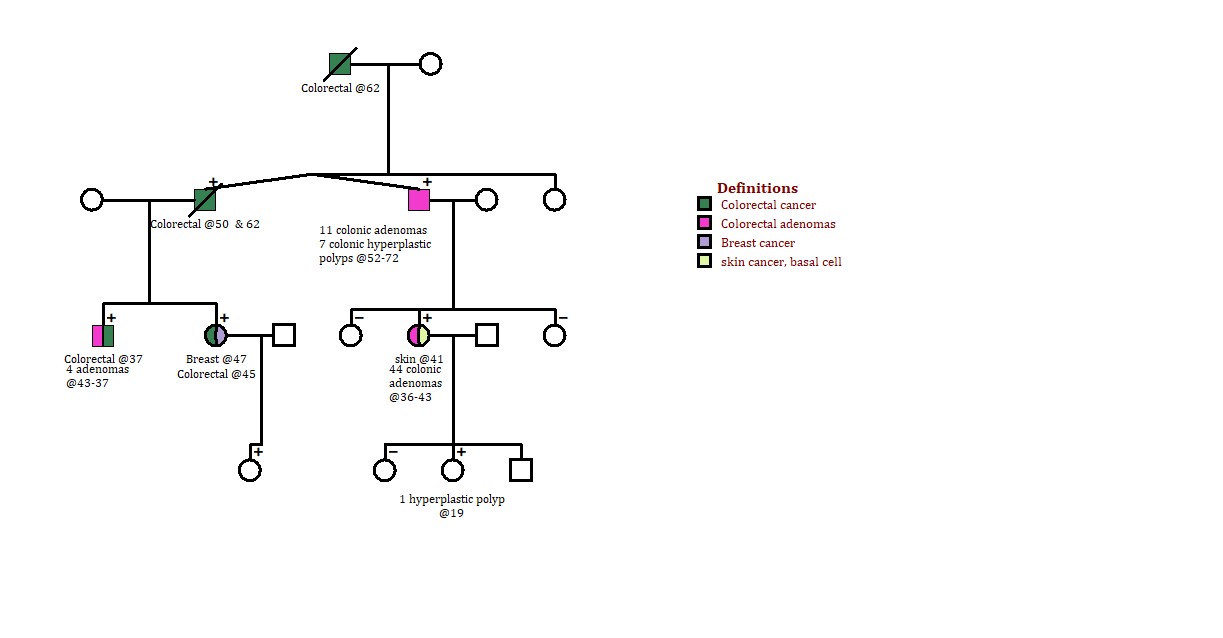
**

**Family D:**

**
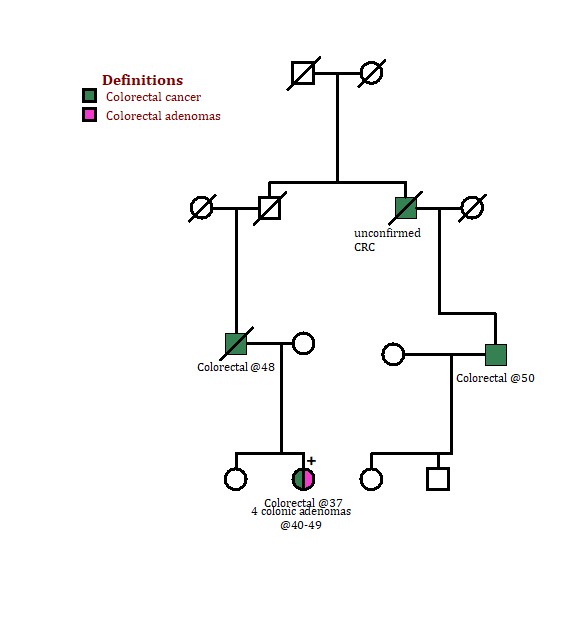
**

**Family E**

**
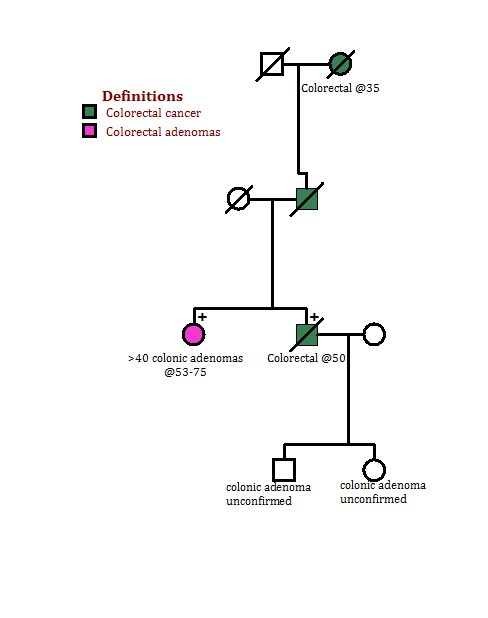
**

**Family I:**

**
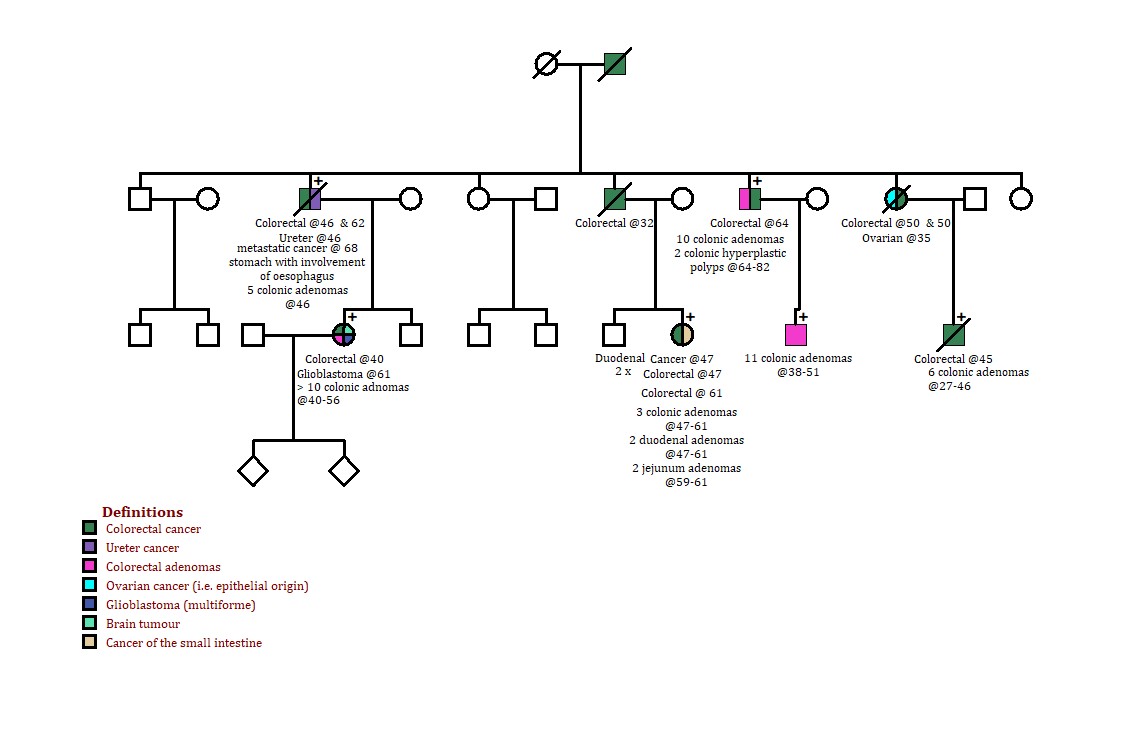
**

**Family K:**

**
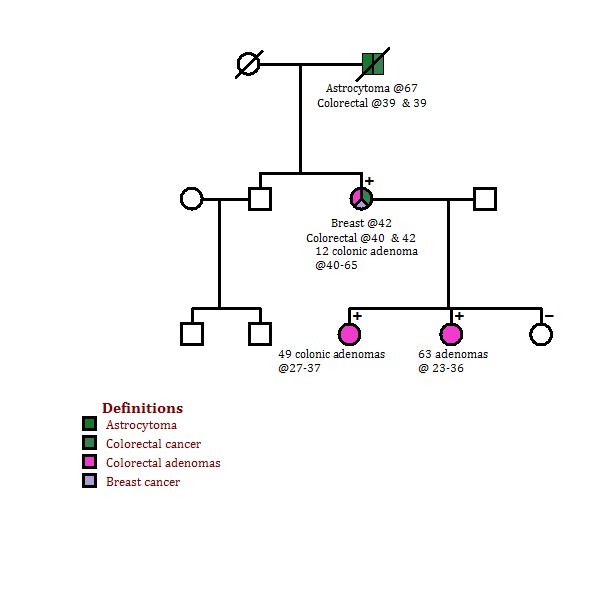
**

**SM7:**


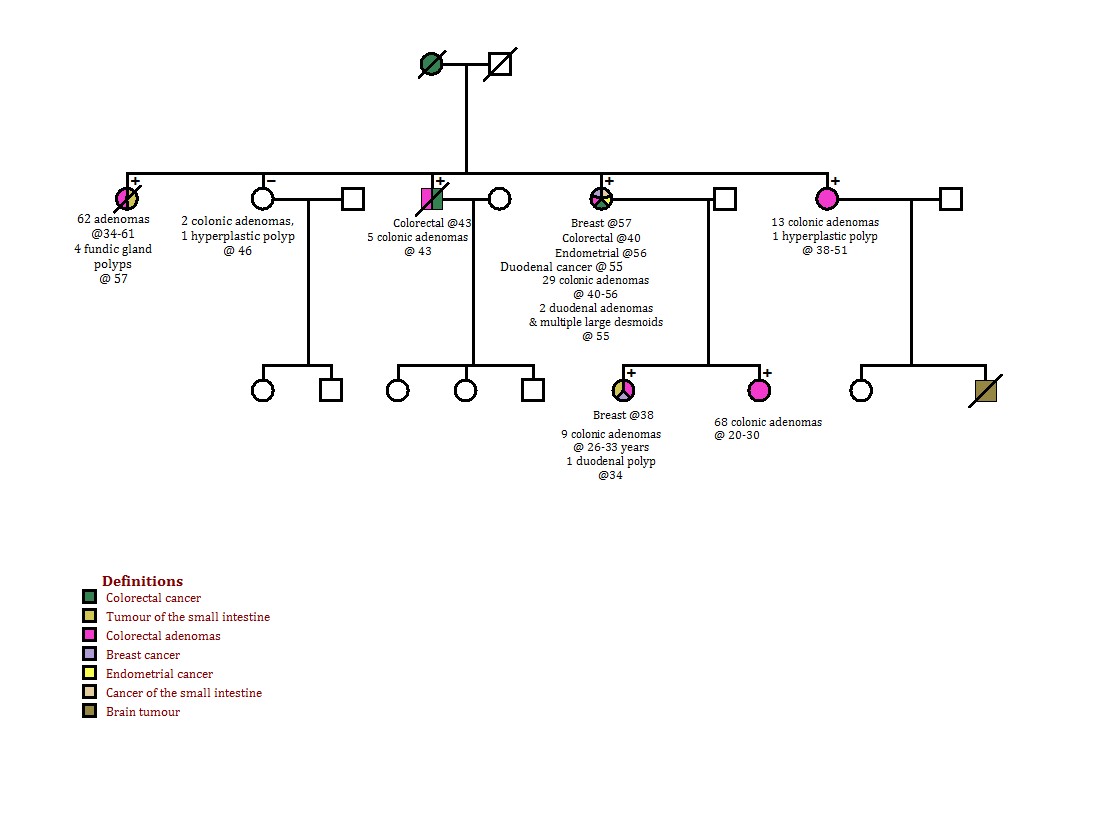


**SM6:**


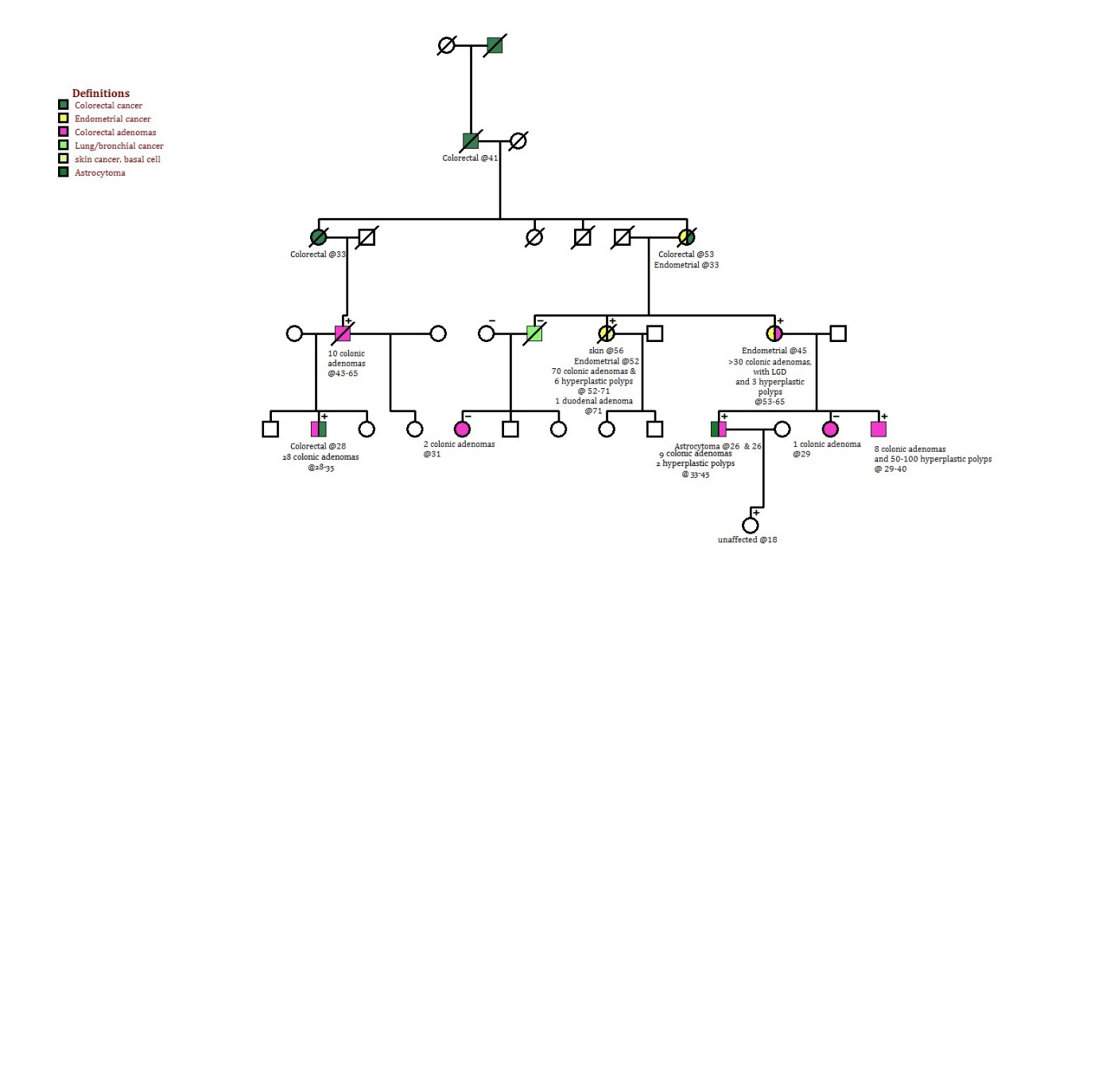


**SM4:**


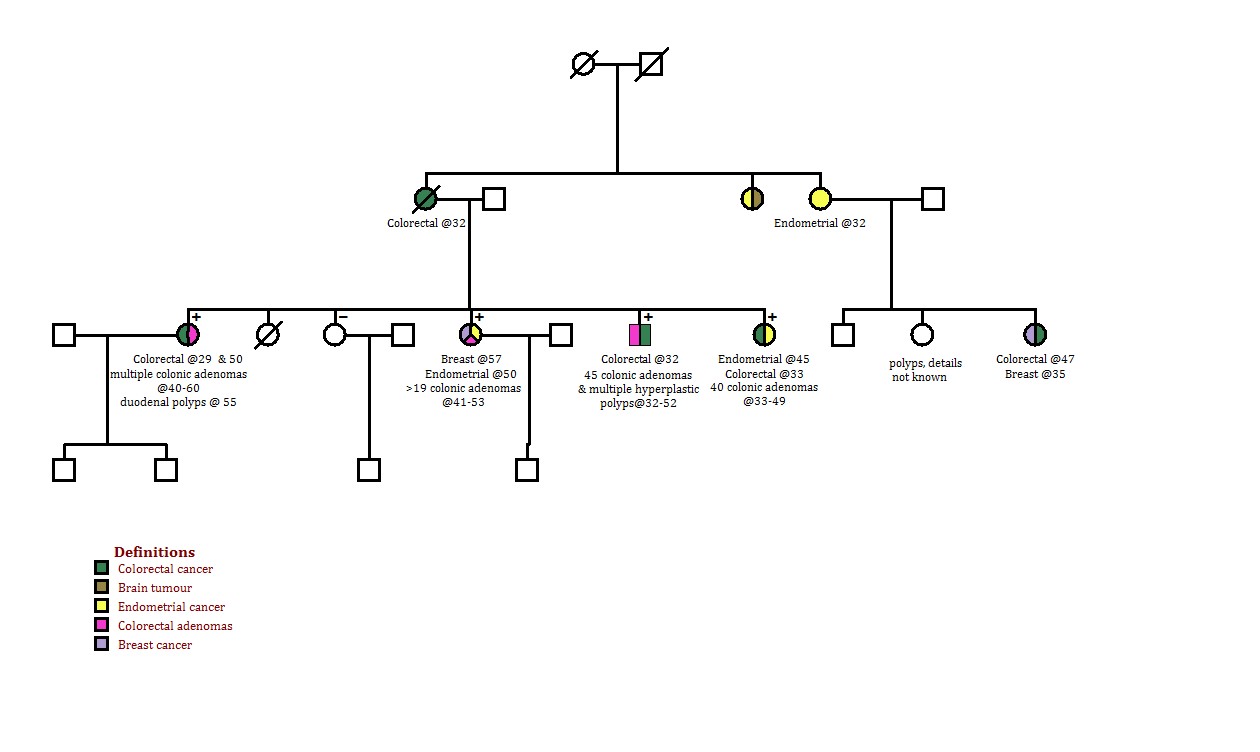


The pedigree of one family is not shown (family L), since full family history was not available. It was known that the proband developed colorectal carcinoma aged 45, and her father was similarly diagnosed at age 28.

Figure 4: Cumulative incidence of breast cancer in all cases


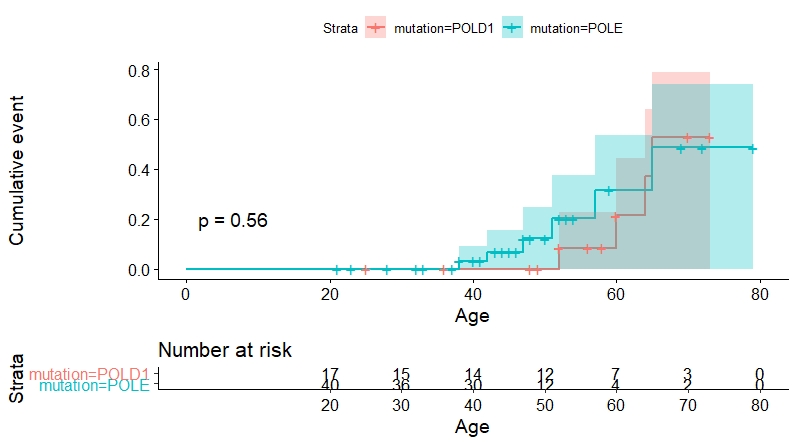


**Supplementary Table 1: HGVS transcript and HGVS predicted protein nomenclature for POLE and POLD1 ED variants identified in the literature review or in patients in CORGI or QUASAR2**

| Gene | Reference sequence | nucleotide change | HGVS_transcript | Reference sequence | Amino acid change | HGVS_Predicted_Protein |
| --- | --- | --- | --- | --- | --- | --- |
| POLD1 | NM_001256849.1 | 931C>T | NM_001256849.1:c.931C>T | NP_001243778.1 | R311C | NP_001243778.1:p.(Arg311Cys) |
| POLD1 | NM_001256849.1 | 946G>A | NM_001256849.1:c.946G>A | NP_001243778.1 | D316N | NP_001243778.1:p.(Asp316Asn) |
| POLD1 | NM_001256849.1 | 946G>C | NM_001256849.1:c.946G>C | NP_001243778.1 | D316H | NP_001243778.1:p.(Asp316His) |
| POLD1 | NM_001256849.1 | 947A>G | NM_001256849.1:c.947A>G | NP_001243778.1 | D316G | NP_001243778.1:p.(Asp316Gly) |
| POLD1 | NM_001256849.1 | 955 T>G | NM_001256849.1:c.955T>G | NP_001243778.1 | C319G | NP_001243778.1:p.(Cys319Gly) |
| POLD1 | NM_001256849.1 | 961G>A | NM_001256849.1:c.961G>A | NP_001243778.1 | G321S | NP_001243778.1:p.(Gly321Ser) |
| POLD1 | NM_001256849.1 | 981C>G | NM_001256849.1:c.981C>G | NP_001243778.1 | P327L | NP_001243778.1:p.(Pro327Leu) |
| POLD1 | NM_001256849.1 | 1040C>T | NM_001256849.1:c.1040C>T | NP_001243778.1 | P347L | NP_001243778.1:p.(Pro347Leu) |
| POLD1 | NM_001256849.1 | 1110C>G | NM_001256849.1:c.1110C>G | NP_001243778.1 | S370R | NP_001243778.1:p.(Ser370Arg) |
| POLD1 | NM_001256849.1 | 1225C>T | NM_001256849.1:c.1225C>T | NP_001243778.1 | R409W | NP_001243778.1:p.(Arg409Trp) |
| POLD1 | NM_001256849.1 | 1276G>A | NM_001256849.1:c.1276G>A | NP_001243778.1 | G426S | NP_001243778.1:p.(Gly426Ser) |
| POLD1 | NM_001256849.1 | 1421T>C | NM_001256849.1:c.1421T>C | NP_001243778.1 | L474P | NP_001243778.1:p.(Leu474Pro) |
| POLD1 | NM_001256849.1 | 1433G>A | NM_001256849.1:c.1433G>A | NP_001243778.1 | S478N | NP_001243778.1:p.(Ser478Asn) |
| POLD1 | NM_001256849.1 | 1519C>T | NM_001256849.1:c.1519C>T | NP_001243778.1 | R507C | NP_001243778.1:p.(Arg507Cys) |
| POLD1 | NM_001256849.1 | 1562G>A | NM_001256849.1:c.1562G>A | NP_001243778.1 | R521Q | NP_001243778.1:p.(Arg521Gln) |
| POLE | NM_006231.3 | 833C>A | NM_006231.3:c.833C>A | NP_006222.2 | T278K | NP_006222.2:p.(Thr278Lys) |
| POLE | NM_006231.3 | 844C>T | NM_006231.3:c.844C>T | NP_006222.2 | P282S | NP_006222.2:p.(Pro282Ser) |
| POLE | NM_006231.3 | 861T>A | NM_006231.3:c.861T>A | NP_006222.2 | D287E | NP_006222.2:p.(Asp287Glu) |
| POLE | NM_006231.3 | 1007A>G | NM_006231.3:c.1007A>G | NP_006222.2 | N336S | NP_006222.2:p.(Asn336Ser) |
| POLE | NM_006231.3 | 1041G>T | NM_006231.3:c.1041G>T | NP_006222.2 | W347C | NP_006222.2:p.(Trp347Cys) |
| POLE | NM_006231.3 | 1055A>C | NM_006231.3:c.1055A>C | NP_006222.2 | Q352P | NP_006222.2:p.(Gln352Pro) |
| POLE | NM_006231.3 | 1089C>A | NM_006231.3:c.1089C>A | NP_006222.2 | N363K | NP_006222.2:p.(Asn363Lys) |
| POLE | NM_006231.3 | 1103A>T | NM_006231.3:c.1103A>T | NP_006222.2 | D368V | NP_006222.2:p.(Asp368Val) |
| POLE | NM_006231.3 | 1231G>C | NM_006231.3:c.1231G>C | NP_006222.2 | V411L | NP_006222.2:p.(Val411Leu) |
| POLE | NM_006231.3 | 1270C>G | NM_006231.3:c.1270C>G | NP_006222.2 | L424V | NP_006222.2:p.(Leu424Val) |
| POLE | NM_006231.3 | 1274A>G | NM_006231.3:c.1274A>G | NP_006222.2 | K425R | NP_006222.2:p.(Lys425Arg) |
| POLE | NM_006231.3 | 1306C>T | NM_006231.3:c.1306C>T | NP_006222.2 | P436S | NP_006222.2:p.(Pro436Ser) |
| POLE | NM_006231.3 | 1336C>T | NM_006231.3:c.1336C>T | NP_006222.2 | R446W | NP_006222.2:p.(Arg446Trp) |
| POLE | NM_006231.3 | 1337G>A | NM_006231.3:c.1337G>A | NP_006222.2 | R446Q | NP_006222.2:p.(Arg446Gln) |
| POLE | NM_006231.3 | 1373A>T | NM_006231.3:c.1373A>T | NP_006222.2 | Y458F | NP_006222.2:p.(Tyr458Phe) |
| POLE | NM_006231.3 | 1378G>A | NM_006231.3:c.1378G>A | NP_006222.2 | V460M | NP_006222.2:p.(Val460Met) |

**Supplementary Table 2. Summary of pathogenicity evidence and clinical manifestations for each germline mutation in the exonuclease domains of *POLE* and *POLD1* that has been reported in the literature, but where we assessed pathogenicity as uncertain**

| **Protein change**  **(nucleotide change)** | **Evidence supporting pathogenicity of variant** | | | | | | | | | **Segregates with affection status** | **Number of carriers (Number of unrelated carriers)** § | **Mean**  **age at diagnosis (range)** | **Number of carriers with Cancer Type** | | | | | | **Number of carriers with** | | **Other cancers reported in carriers** | **Ref** |
| --- | --- | --- | --- | --- | --- | --- | --- | --- | --- | --- | --- | --- | --- | --- | --- | --- | --- | --- | --- | --- | --- | --- |
|  | **Structure** | **Conserved in POLD1-POLE alignment** | **Grantham Score** | **SIFT** | **Polyphen** | **Phylop score*** | **Significantly greater # of mutations yeastψ** | **Biochemical evidence for functional change**‡ | **GnomAD frequency** |  |  |  | **Colorectal** | **Endometrial** | **Breast** | **Duodenal** | **Ovarian** | **Brain** | **Duodenal adenomas** | **Colonic adenomas** |  |  |
| **Polε** | | | | | | | | | | | | | | | | | | | | | | |
| NP_006222.2:p.(Pro282Ser) | Exo 1 motif | partially | 74 | 0.003 | 1 | 9.95 | NR | NR | 6 x 10^-5^ | NR | 1(1) | 55 | 0 | 0 | 0 | 0 | 0 | 0 | 0 | 0 | cutaneous malignant melanoma (N=1), merkel cell carcinoma (N=1) | [8] |
| NP_006222.2:p.(Asp287Glu) | Flanking Exo 1 motif (2 bases away) | partially | 45 | 0.002 | 1 | 1.19 | No (Sup. Fig 2) | NR | 8.6 x 10^-4^ | No | 13 (8) | 49  (34-73) | 3 | 2 | 1 | 0 | 0 | 0 | 0 | 0 | cutaneous malignant melanoma(N=6), non Hodgkins lymphoma (N=1), squamous cell carcinoma (N=1) | [1-3, 8] |
| NP_006222.2:p.(Asn336Ser) | Outside exo motifs | partially | 46 | 0 | 1 | 7.98 | NR | NR | 0.0027 | Not tested | 1 (1) | Not reported | 0 | 1 | 0 | 0 | 0 | 0 | 0 | 0 | None | [2] |
| NP_006222.2:p.(Trp347Cys) | Outside exo motifs | fully | 215 | 0.001 | 0.37 | 5.78 | Yes [8] | NR | 3.18 x 10^-5^ | No^#^ | 11 (1) | 49  (14-70) | 0 | 0 | 0 | 0 | 0 | 0 | NA | NA | Prostate (N=1), cutaneous and uveal malignant melanoma (N=4) | [8] |
| NP_006222.2:p.(Gln352Pro) | Outside exo motifs | partially | 76 | 0.24 | 0.98 | 7.76 | NA | NA | 3.51 x 10^-5^ | No^##^ | 3 (2) | 55 | 1 |  |  |  |  |  |  |  | cutaneous malignant melanoma (N=1). | [8, 9] |
| NP_006222.2:p.(Lys425Arg) | Within Exo IV | partially | 76 | 0 | 1 | 7.96 | NA | NA | 3.1 x 10^-5^ | Not tested | 2 (2) | 55 (age of CM case, age of CRC case not reported) | 1 |  |  |  |  |  |  |  | cutaneous malignant melanoma (N=1). | [8, 10] |
| NP_006222.2:p.(Pro436Ser) | Within ExoV motif | partially | 74 | 0 | 1 | 9.95 | NA | NA | 0 | de novo | 1(1) | 31 | 1 | 0 | 0 | 0 | 0 | 0 | 1 | 1 | None | [11] |
| NP_006222.2:p.(Arg446Gln) | Flanking Exo V (8 bases away) | partially | 43 | 0.073 | 0.99 | 7.87 | NA | NA | 3.59x 10^-5^ | NA | 3(3) | 45 | 0 | 1 | 0 | 0 | 0 | 0 | 0 | 1 | cutaneous malignant melanoma (N=1) | [6, 8, 11] |
| NP_006222.2:p.(Arg446Trp) | Flanking Exo V (8 bases away) | partially | 101 | 0.003 | 1 | 4.86 | NA | NA | 2.8 x 10^-4^ | NA | 2(2) | 57 | 1 | 1 | 0 | 0 | 0 | 0 | 0 | 0 | Soft tissue (N=1) | [2, 3] |
| NP_006222.2:p.(Val460Met) | Within Exo III | partially | 21 | 0 | 1 | 7.77 | NA | NA | 8.79 x 10^-6^ | Yes | 2 (1) | 52 (CMM case diagnosed at 29, CRC case at 74) | 1 |  |  |  |  |  |  |  | cutaneous malignant melanoma aged 29 (N=1). Case with CRC also had prostate cancer at 70 and AML at 83 | [8] |
| **Polδ** | | | | | | | | | | | | | | | | | | | | | | |
| NP_001243778.1:p.(Arg311Cys) | Flanking ExoI motif ( 1 base away) | partially | 180 | 0 | 0.99 | 0.63 | NA | NA | 1.46 x 10^-5^ | NA | 1 (1) | ≤70 | 0 | 1 | 0 | 0 | 0 | 0 | 0 | 0 |  | [6] |
| NP_001243778.1:p.(Cys319Gly) | Exo 1 motif | partially | 159 | 0 | 0.99 | 5.45 | NA | NA | 4.14 x 10^-6^ | No | 1 | 44 | 1 | 0 | 0 | 0 | 0 | 0 | 0 | 1 |  | [12] |
| NP_001243778.1:p.(Gly321Ser) | Exo1 motif | partially | 56 | 0.009 | 0.95 | 7.17 | NA | NA | 3.5 x 10^-4^ | No | 3 (3) | 40 | 3 | 0 | 0 | 0 | 0 | 0 | 0 | 2 |  | [1, 12] |
| NP_001243778.1:p.(Pro347Leu) | Outside exo motifs | partially | 98 | 0.08 | 0.01 | 0.06 | NA | NA | 4.12 x 10^-5^ | NR | 1(1) | 50 | 1 | 0 | 0 | 0 | 0 | 0 | 0 | 0 |  | [9] |
| NP_001243778.1:p.(Arg409Trp) | Flanking Exo II motif (3 bases away) | fully | 101 | 0 | 1 | 1.12 | NA | NA | 3.98 x 10^-6^ | NA | 1 (1) | 32 | 1 | 0 | 0 | 0 | 0 | 0 | 0 | 1 |  | [13] |
| NP_001243778.1:p.(Ser370Arg) | Outside exo motifs | partially | 110 | 0.04 | 0.41 | 5.34 | NA | NA | 0 | NA | 1 (1) | 32 | 1 |  |  |  |  |  |  | 1 |  | [14] |
| NP_001243778.1:p.(Gly426Ser) | Outside exo motifs | partially | 56 | 0.43 | 0.04 | 2.44 | NA | NA | 6.49 x 10^-5^ | Not reported | 2 (2) | 55 | 1 |  |  |  |  |  |  | 1 |  | [9, 14] |
| NP_001243778.1:p.(Arg507Cys) | ExoIII | partially | 180 | 0 | 1 | 5.20 | NA | NA | 0 | de novo | 1 (1) | 25 | 0 | 0 | 0 | 0 | 0 | 0 | 0 | 0 | Mandibular hypoplasia, deafness and progeroid features (MDPL syndrome)(N=1) | [15] |
| NP_001243778.1:p.(Arg521Gln) | Flanking Exo III (2 bases away) | partially | 43 | 0.05 | 0.82 | 8.76 | NA | NA | 2.2 x 10^-4^ | Not reported | 1 (1) | 48 | 1 |  |  |  |  |  |  |  |  | [13] |

Variants shown in this table lack in vitro evidence of pathogenicity from either yeast mutation rate assays or biochemical assays of exonuclease function and have no evidence of so-segregation of mutation and affection status. The exception being W347C, which displayed a mutator phenotype in yeast but did not co-segregate with disease status in families.

A or P, adenomas or polyps; BrC, breast cancer; CRC, colorectal cancer; DuC, duodenal carcinoma; EC, endometrial cancer; GBM, glioblastoma; NR, not reported;

OC, ovarian cancer; ODG, oligodendroglioma. SIFT, Polyphen and PhyloP (100 way vertebrate) scores were obtained from dbNSFPv33a ‡Data from functional studies of B family polymerases. §

Reported as of April 2018. Mean age at diagnosis in years refers to cancer or adenoma diagnosis, whichever was earliest. ^¶^ Functional studies of the corresponding residue in Pol ε.

#Six mutation carriers were unaffected, ^##^ one mutation carrier was unaffected

Ψ Significantly greater number of mutations in strain with the equivalent mutation when compared to reference strain

**Supplementary Table 3: Cancer types diagnosed in cases with presumed pathogenic *POLE* exonuclease domain mutations and known screening and surgery undergone**

| Family ID Mutation | Patient ID | Sex | Cancers | | | | | | | Polyps | | | Micro-satellite status | Ref | Screening undergone | Surgery undergone |
| --- | --- | --- | --- | --- | --- | --- | --- | --- | --- | --- | --- | --- | --- | --- | --- | --- |
|  |  |  | Colo-rectal (age) | Endo-metrial (age) | Breast (age) | Duodenal/  Stomach (age) | Brain  Type  (age) | Ovarian (age) | Other (age) | Duodenal adenomas (age) | colonic adenomas (age) | colonic  hyper-plastic (age) |  |  |  |  |
| Fig1: Thr278Lys | II:1 | M | 1 (54) |  |  |  |  |  |  |  | >80 (54) |  |  | [16] |  |  |
| Fig1: Thr278Lys | II:2 | F | 4 (2 x 52, 1 x 53, 1 x 54) |  |  |  |  |  |  |  | >100 (54) |  |  | 15] |  |  |
| Fig1: Thr278Lys | II:3 | F | 4 (2 x 39, 2 x 57) |  | 1 (51) |  |  |  |  |  | 35 (59) |  |  | 15] |  |  |
| Fig1: Thr278Lys | II:4 | M |  |  |  |  |  |  |  |  | >40 (46) |  |  | 15] |  |  |
| Fig1:  Asn363Lys | II:2 | F | 1 (43) | 1(50) |  |  |  | 1 (50) |  |  |  |  | MSS* | [17] |  |  |
| Fig1:  Asn363Lys | II:4 | M | 1 (56) |  |  |  |  |  |  |  |  |  | MSS* | [17] |  |  |
| Fig1:  Asn363Lys | II:7 | M | 1 (28) |  |  |  |  |  |  |  |  |  | MSS* | [17] |  |  |
| Fig1:  Asn363Lys | II:9 | F | 2  (57, 78) |  |  |  |  | 1 (45) | pancreatic |  |  |  |  | [17] |  |  |
| Fig1:  Asn363Lys | II:11 | F |  | 1 (45) |  |  |  | 1 (45) |  |  |  |  |  | [17] |  |  |
| Fig1:  Asn363Lys | III:2 | M | 1 (35) |  |  |  |  |  |  |  |  |  |  | [17] |  |  |
| Fig1:  Asn363Lys | III:4 | F | 2  (47, 53) |  |  |  |  |  |  |  | adenomas (not stated) |  |  | [17] |  |  |
| Fig1:  Asn363Lys | III:6 | M | 1 (55) |  |  |  |  |  |  |  |  |  |  | [17] |  |  |
| Fig1:  Asn363Lys | III:9 | F | 1 (40) |  |  |  |  |  |  |  | adenomas (not stated) |  |  | [17] |  |  |
| Fig1:  Asn363Lys | III:10 | M |  |  |  |  | giant cell glioblast-oma (28) |  |  |  |  |  |  | [17] |  |  |
| Fig1:  Asn363Lys | IV:2 | M | 1 (38) |  |  |  |  |  |  |  |  |  |  | [17] |  |  |
| Fig1:  Asn363Lys | IV:3 | M |  |  |  |  |  |  |  |  | adenomas (not stated) |  |  | [17] |  |  |
| new confirmed case  Asn363Lys | IV:5 | M |  |  |  |  |  |  |  |  | adenomas (34) |  |  | New case from family reported in [17] |  |  |
| new confirmed case  Asn363Lys | III:11 | F |  |  |  |  |  |  |  |  | adenomas (44) |  |  | New case from family reported in [17] |  |  |
| new confirmed case  Asn363Lys | IV:4 | F |  |  |  |  | Brain tumour (35) |  |  |  |  |  |  | New case from family reported in [17] |  |  |
| Fig1:  Asn363Lys | II:10 | M | 1 (67) |  |  |  |  |  |  |  | adenomas (not stated) |  |  | [18] |  |  |
| Fig1:  Asn363Lys | III:3 | M | 1 (41) |  |  |  | giant cell glio-bastoma (45) |  |  |  |  |  | MSS | [18] |  |  |
| Fig1:  Asn363Lys | III:4 | F | 1 (37) |  |  |  | giant cell glio-blastoma (52) |  |  | 1 adenoma (not stated) | 5 adenomas (not stated) |  | MSS | [18] |  |  |
| Fig1:  Asn363Lys | III:5 | M | 1 (52) |  |  |  |  |  | prostate (51) |  | 5 adenomas (not stated) |  | MSS | [18] |  |  |
| Fig1:  Asn363Lys | III:10 | M | 0 (52) |  |  |  |  |  |  |  | adenomas (not stated) |  |  | [18] |  |  |
| Fig1:  Asn363Lys | III:11 | F | 0 (50) |  |  |  |  |  |  |  | adenomas (not stated) |  |  | [18] |  |  |
| Fig1:  Asn363Lys | III:16 | M | 1 (52) |  |  | duodenal(52) synchronous with CRC |  |  |  |  | adenomas (19) |  | MSS | [18] |  |  |
| Fig1:  Asn363Lys | III:17 | F | 0 (50) |  |  |  |  |  |  |  | adenomas (not stated) |  |  | [18] |  |  |
| Fig1:  Asn363Lys | III:18 | M | 1 (57) |  |  | duodenal (48) |  |  |  |  |  |  |  | [18] |  |  |
| Fig1:  Asn363Lys | IV:5 | M |  |  |  | Gastric carcinoma (36) |  |  |  |  | >50 incl intraepithelial adenocarcinoma |  |  | [18] |  |  |
| Fig1:  Asn363Lys | IV:15 | M | 1 (23) |  |  |  | Glio-blastoma (30) |  |  |  | 3 adenomas (not stated) |  | MSS | [18] |  |  |
| Data Supp  Asp368Val | 2146201 | F | 1 (47) |  |  |  |  |  |  |  |  |  | MSS | [9] |  |  |
| B  Leu424Val | III.1 | F | 2 (48) |  |  |  |  |  |  |  | 3  (43-70) | 1 | - | [14] | colonoscopy and hysteroscopy | right hemi-colectomy |
| B  Leu424Val | IV.2 | M | 1 (29) |  |  |  |  |  |  |  | 19  (29-45) |  | - | [14] | colonoscopy |  |
| C  Leu424Val | II.2 | M | 1 (50) |  |  | duodenal (63) |  |  | Metastatic CRC in mesentery (63) |  |  |  | MSS (CRC) | [14] |  | CRC resection, whipples |
| C  Leu424Val | II.3 | M |  |  |  |  |  |  |  |  | 11  (52-72) | 7 | - | [14] | colonoscopy |  |
| C  Leu424Val | III.1 | M | 1 (37) |  |  |  |  |  |  |  | 4  (43-47) |  | - | [14] | colonoscopy | right hemicolectomy |
| C  Leu424Val | III.2 | F | 1 (45) |  | 1 (47) |  |  |  |  |  |  |  |  | New CORGI |  | right hemicolect-omy |
| C  Leu424Val | III.4 | F |  |  |  |  |  |  | basal cell carcinoma (41) |  | 44  (36-43) |  |  | New CORGI | colonoscopy and hysteroscopy (endometrial dysplasia found) |  |
| C  Leu424Val | IV:2 | F |  | unaffected aged 24 | | | | | |  |  |  |  | New CORGI | colonoscopy |  |
| C  Leu424Val | IV:3 | F |  |  |  |  |  |  |  |  | 1 (19) |  |  | New CORGI | colonoscopy, renal ultrasound |  |
| D  Leu424Val | IV.2 | F | 1 (37) |  |  |  |  |  |  |  | 4  (40-49) |  | - | [14] | colonoscopy and hysteroscopy |  |
| E  Leu424Val | III.1 | F |  |  |  |  |  |  |  | 1 (76) | 45 (53) |  | - | [14] | colonoscopy, gastroscopy and duodenal biopsy | colectomy |
| E  Leu424Val | III.2 | M | 1 (50) |  |  |  |  |  |  |  |  |  |  | [14] |  | CRC resection |
| I  Leu424Val | II.3 | M | 2 (46)  1 (62) |  |  | stomach* |  |  | ureter |  | 5  (64-68) |  | - | [14] | colonoscopy | Nephrectomy and right hemi colectomy for cancers diagnosed at 46. |
| I  Leu424Val | II.9 | M | 1 (64) |  |  |  |  |  |  |  | 10  (65-66) | 2(64-82) | MSS (10 ads) | [14] | colonoscopy | right hemi-colectomy |
| I  Leu424Val | III.4 | F | 1 (40) |  |  |  | Glio-blastoma (61) |  |  |  | >10  (40-56) |  | MSS (1 ad) | [14] | colonoscopy | Hysterectomy following identification of severe complex hyperplasia with moderate cellular atypia & bilateral salpingoophorectomy following identification of adhesions. Colectomy with ileorectal anastomosis following CRC aged 40 |
| I  Leu424Val | III.9 | F | 2 (47)  1 (61) |  |  | duodenal (47) |  |  |  | 2 (47-61), 2 jejunum adenomas(59-61) | 3 (47-61) |  | MSI | [14] | Colonoscopy, gastroscopy. | Left hemi-colectomy for first two CRCs, duodenal resection, duodectomy and distal gastrectomy. Resection for third CRC. Hysterectomy aged 61. |
| I  Leu424Val | III.10 | M |  |  |  |  |  |  |  |  | 11  (39-51) |  | - | [14] | colonoscopy |  |
| New case from family I Leu424Val | III.11 | M | 1 (45) |  |  |  |  |  |  |  | 6 (27-45) |  |  | New case from family reported in  [14] |  |  |
| K  Leu424Val | II.3 | F | 2 (40,42) |  | 1 (42) |  |  |  |  |  | 12  (40-65) |  | MSS | [14] | Colonoscopy |  |
| K  Leu424Val | III.3 | F |  |  |  |  |  |  |  |  | 49  (27-38) |  | MSS (1 ad) | [14] | Colonoscopy |  |
| K  Leu424Val | III.4 | F |  |  |  |  |  |  |  |  | 63  (24-40) |  | MSS | [14] | Colonoscopy |  |
| L  Leu424Val | II.1 | F | 1 (45) |  |  |  |  |  |  |  |  |  |  | [14] | Colonoscopy |  |
| SM7  Leu424Val | II.1 | F |  |  |  |  |  |  |  |  | 62  (34-61) |  | - | [14] | colonoscopy, gastroscopy, hysteroscopy, Ovarian cystectomy | Hysterectomy (endo-metriosis and endometriotic cyst) |
| SM7  Leu424Val | II.4 | M | 1 (43) |  |  |  |  |  |  |  | 5(43) |  | MSS (2 ads) | [14] | Colonoscopy |  |
| SM7  Leu424Val | II.6 | F | 1 (40) | 1 (56) | 1(57) | duodenal (55) |  |  |  | 2 (55) | 29  (40-56) |  | MSS (CRC, 17 ads) | [14] | Colonoscopy | multiple cancer resections and colectomy |
| SM7  Leu424Val | II.8 | F |  |  |  |  |  |  |  |  | 13  (38-51) | 1 | MSS (1 ad) | [14] | Colonoscopy |  |
| SM7  Leu424Val | III.6 | F |  |  | 1 (38) |  |  |  |  | 1 (34) | 9  (26-33) |  | - | [14] | Colonoscopy, Gastroscopy |  |
| SM7  Leu424Val | III.7 | F |  |  |  |  |  |  |  |  | 68  (20-30) |  | - | [14] | Colonoscopy |  |
| M  Leu424Val | II.1 | M | 1 (48) |  |  |  |  |  | oesophageal cancer (69), bone metastasis consistent with pancreatic origin (70)* |  | 1 (48) |  |  | New CORGI | Colonoscopy | Abdomino-perineal resection |
| M  Leu424Val | II.3 | F |  |  |  |  |  |  |  |  | 1 large (45) | 2 |  | New CORGI | colonoscopy |  |
| M  Leu424Val | II.4 | F | 1 (60) |  | 1 (65) |  |  |  |  |  | 4 (44-60) |  |  | New CORGI | colonoscopy, gastroscopy- multiple benign polyps in stomach including fundal gland polyps | right hemi-colectomy |
| N  Leu424Val | IV.2 | F | 2 (71) | 1 (53) |  | 1 (76) |  |  | ureter (71) |  | 4 (71) |  |  | New CORGI | colonoscopy |  |
| N  Leu424Val | V.1 | M | 1 (46) |  |  |  |  |  | Basal cell carcinoma (48) | 1 > 10mm (51) | 8 (46-51) |  | MSS | New CORGI | colonoscopy | right hemi-colectomy |
| N  Leu424Val | VI.2 | M |  |  |  |  |  |  |  | 1 (17) | 1 (15) |  |  | New CORGI | colonoscopy |  |
| QUASAR2 pt1 Leu424Val | NA | M | 1 (46) |  |  |  |  |  |  |  |  |  |  | Q2 |  | resection |
| QUASAR2 pt2  Leu424Val | NA | M | 1 (37) |  |  |  |  |  |  |  |  |  |  | Q2 |  | resection |
| Figure 1A  Leu424Val | III.6 | F | 1 (28) |  |  |  | anaplastic oligo-dendro-glioma (not stated) |  |  |  | 39 (28-30) | 2  (28) |  | [19] | Colonoscopy |  |
| Family 1  Leu424Val | (PT1) | F | 1 (40) | 1 (50) |  |  |  |  |  |  | 30  (not stated) |  | MSI (CRC) MSS (EC) | [20] | Colonoscopy |  |
| Family 1  Leu424Val | PT2 | M | 1 (30) |  |  |  | Astro-cytoma  (not stated) |  | Neurofibromatosis and associated astrocytoma -from non-carrier side of family. (15) |  |  |  | MSI | [20] | Colonoscopy |  |
| Family 2  Leu424Val | PT3 | U | 1 (34) |  |  |  |  |  |  |  | multiple (>10) (34) |  | MSI (CRC), MSS (1 AP) | [20] | Colonoscopy |  |
| Family 3  Leu424Val | PT4 | U | 1 (33) |  |  |  |  |  |  |  | polyps (33) |  | MSS | [20] | Colonoscopy |  |
| F156/F381  Leu424Val | F156_III .5 | U | 1 (27) |  |  |  |  |  | Pilomatri-coma (not stated) | adenomas +4 jejunum adenomas (not stated) | 51-100 (27) |  | - | [11] | Colonoscopy, gastroscopy |  |
| F156/F381  Leu424Val | F156_III .1 | U |  |  |  |  |  |  |  | adenomas (not stated) | >100 (35) | >50 | - | [11] | Colonoscopy, gastroscopy |  |
| F156/F381  Leu424Val | F156_III .3 | U | 1 (42) |  |  |  |  |  |  | adenomas (not stated) | multiple (>10) (32) |  | - | [11] | Colonoscopy, gastroscopy |  |
| F156/F381  Leu424Val | F156_III .4 | U | 5 (30) |  |  |  |  |  |  |  | 51-100 (30) | >50 | - | [11] | Colonoscopy, gastroscopy (gastric fundic gland polyps ) |  |
| F156/F381  Leu424Val | F381_II .6 index | M | 4 (36) |  |  |  |  |  |  | adenomas (not stated) | 51-100 (36) |  | MSS | [11] | Colonoscopy, gastroscopy |  |
| F156/F381  Leu424Val | F381_III .1 | M | 1 (36) |  |  | duodenal (45) |  |  | sarcoidosis |  | 21-50 (34) |  | MSS (duodenal cancer) | [11] | Colonoscopy |  |
| F156/F381  Leu424Val | F381_III .2 | F |  |  |  |  |  |  |  |  | 21-50 (37) |  | - | [11] | Colonoscopy |  |
| F156/F381  Leu424Val | F381_III .5 | F |  |  |  |  | Glioblastoma (not stated) |  |  | adenomas (not stated) | 11-20 (32) |  | - | [11] | Colonoscopy, gastroscopy (gastric fundic gland polyps) |  |
| F354 Leu424Val | II:5 (index) | not stated | 3 (27,38, 39) |  |  |  |  |  | neuroendo- crine carcinoma | adenomas (not stated) | 51-100 (27) |  | MSS | [11] | Colonoscopy |  |
| F354 Leu424Val | III:5 | not stated |  |  |  |  |  |  |  |  | 11-20 adenomas and HPs (16) |  |  | [11] | Colonoscopy |  |
| F1505  Leu424Val | index | not stated | 1 (38) |  |  |  |  |  | 1 lipoma |  | 51-100 (38) |  | MSS (IHC no loss) | [11] | Colonoscopy |  |
| H1427  Leu424Val | III:1 (index) | not stated | 2 (46,48) |  |  |  |  |  |  |  | <10 (46) |  | MSS (IHC no loss) | [11] | Colonoscopy |  |
| H1427  Leu424Val | II:2 | not stated |  |  |  |  |  |  |  |  | <10 (63) |  |  | [11] | Colonoscopy |  |
| H1427  Leu424Val | III:8 | presumed female | 3 (2 x 45, 1 x 53) |  |  |  |  | 1 (33) |  |  | 21-50 adenomas and HPs (not stated) |  |  | [11] | Colonoscopy |  |
| 16393201  Leu424Val | 16393201 | M | 1 (43) |  |  |  |  |  |  |  | 3 | 2 |  | [9] | Colonoscopy |  |
| 9811201  Leu424Val | 9811201 | F | 1 (45) |  |  |  |  |  |  |  | adenomas (not stated) |  |  | [9] | Colonoscopy |  |
| 9811201  Leu424Val | 9811201 paternal cousin | M | 1 (46) |  |  |  |  |  |  |  | adenomas (not stated) |  |  | [9] | Colonoscopy |  |
| Wimmer  Val411Leu |  | M | 1 (14) |  |  |  |  |  |  | adenomas (14) | adenomas (14) |  | MSS | [21] | Colonoscopy |  |
| ES family  Tyr458Phe | (III.2) | M | 1 (63) |  |  |  |  |  |  |  |  |  |  | [22] | Colonoscopy |  |
| ES family  Tyr458Phe | (IV:8) | M |  |  |  |  |  |  | Pancreatic (46) |  |  |  |  | [22] | Colonoscopy |  |
| ES family  Tyr458Phe | (IV:9) | F | 1 (48) |  |  |  |  | 2(40) |  |  | multiple (not stated) |  |  | [22] | Colonoscopy |  |
| ES family  Tyr458Phe | (IV:10) | F |  |  |  |  |  |  |  |  | multiple (not stated) |  |  | [22] | Colonoscopy |  |
| ES family  Tyr458Phe | (IV:13) | F | 1 (43) |  |  |  |  |  |  |  | multiple (not stated) |  |  | [22] | Colonoscopy |  |
| ES family  Tyr458Phe | (IV:15) | M | 1 (58) |  |  |  |  |  |  |  |  |  |  | [22] | Colonoscopy |  |
| ES family  Tyr458Phe | (IV:17) | M | 1 (42) |  |  | Jejunum cancer (54), duodenal cancer (57) |  |  |  |  | multiple (35) |  |  | [22] | Colonoscopy | Colectomy with ileorectal anastomosis. |
| ES family  Tyr458Phe | (IV:20) | M | 1 (38) |  |  |  |  |  |  |  |  |  |  | [22] | Colonoscopy |  |
| ES family  Tyr458Phe | (IV:21) | M | 1 (56) |  |  |  |  |  |  |  | adenomas (not stated) |  |  | [22] | Colonoscopy |  |
| ES family  Tyr458Phe | (V:4) | M |  |  |  |  |  |  |  |  | multiple (late twenties) |  |  | [22] | Colonoscopy |  |
| ES family  Tyr458Phe | (V:5) | M |  |  |  |  |  |  |  |  | multiple (late twenties) |  |  | [22] | Colonoscopy |  |
| ES family  Tyr458Phe | (V:8) | F |  |  |  |  |  |  |  |  | multiple (late twenties) |  |  | [22] | Colonoscopy |  |
| Validation  Tyr458Phe | index | M | 1(44) |  |  | duodenum (59) |  |  |  | 1 (not stated) |  |  |  | [22] | Colonoscopy |  |
| Validation  Tyr458Phe | son | M | 1 (42) |  |  |  |  |  |  |  |  |  |  | [22] | Colonoscopy |  |
| Validation  Tyr458Phe | brother | M |  |  |  |  |  |  |  |  | adenomas (34) |  |  | [22] | Colonoscopy |  |

* metastatic cancer. MSS* Manuscript states that tumours from 3 individuals in the family were MSS

**Supplementary Table 4: Cancer types diagnosed in cases with presumed pathogenic *POLD1* exonuclease domain mutations and known screening and surgery they have undergone**

| Family ID  Mutation  Reference | Patient ID | Sex |  | Cancers | | | | | | | Polyps | | |  |  |  |  |
| --- | --- | --- | --- | --- | --- | --- | --- | --- | --- | --- | --- | --- | --- | --- | --- | --- | --- |
|  |  |  | Colo-rectal (age) | | Endo-metrial (age) | Breast (age) | Duodenal/  Stomach (age) | Brain  type  (age) | Ovarian  (age) | Other  (age) | duodenal (age) | colonic adenomas (age) | colonic hyper-plastic (age) | Micro-satelite status | Ref | Screening undergone | Surgery undergone |
| Validation case  Pro327Leu | Validation case | F |  | |  |  |  |  |  |  |  | 10 (70) |  |  | [14] | Colonoscopy |  |
| Fam2  Asp316Gly | II:2 | F |  | | 1 (57) | 2 (65,75) |  |  |  |  |  |  |  | MSS | [13] |  |  |
| Fam2  Asp316Gly | III:2 | F | 1(44) | | 1 (54) |  |  |  |  |  |  | 3 (56) |  | MSS | [13] |  |  |
| Fam1  Asp316His | III:7 | F |  | |  | 1 (64) |  |  |  |  |  | 13 (not stated) |  |  | [13] |  |  |
| Fam1  Asp316His | III:8 | M | 1 (58) | |  |  |  |  |  | Mesothelioma (aged 65) and angio-myolipoma |  | 4 (not stated) |  | MSS | [13] |  |  |
| Family O  Asp316Asn | IV:2 | F |  | | 1 (54) |  |  |  |  |  |  | 5 (58) |  |  | New CORGI |  |  |
| Family O  Asp316Asn | IV:3 | F |  | |  | 1 (52) |  |  |  |  |  |  |  |  | New CORGI |  |  |
| Fig. 1B  Leu474Pro | (II.2) | F |  | | 1 (52) |  |  |  |  |  |  |  |  |  | [19] |  |  |
| Fig. 1B  Leu474Pro | (II.6) | F | 1 (33) | | 1 (56) |  |  |  |  |  |  | 0 (56) |  |  | [19] |  |  |
| Fig. 1B  Leu474Pro | (III.1) | F | 1 (36) | |  |  |  |  |  | Gastro-intestinal stromal tumour of the large bowel (36) |  | 0(36) |  |  | [19] | Follow up (presumed colonoscopy) | resection |
| Fam 4  Leu474Pro | III:1 | F |  | |  |  |  |  |  |  |  | 2(48) |  |  | [13] |  |  |
| Fam 4  Leu474Pro | III:2 | M | 1(50) | |  |  |  |  |  |  |  |  |  |  | [13] |  |  |
| Fam 4  Leu474Pro | IV:1 | F | 1 (23) | |  |  |  |  |  | Benign esophageal tumour, gastric polyps |  |  |  |  | [13] |  |  |
| Family P  Leu474Pro | III:1 | M | 2 (21,31) | |  |  |  |  |  |  |  |  |  |  | New CORGI |  | Pan-proctocolectomy |
| Family P  Leu474Pro | IV:3 | M |  | |  |  |  |  |  |  |  | 9 (19) |  |  | New CORGI | Colonoscopy | subtotal colectomy |
| SM6  Ser478Asn | IV:2 | M |  | |  |  |  |  |  |  |  | 10 (43-65) |  | MSS | [14] | colonoscopy |  |
| SM6  Ser478Asn | IV:6 | F |  | | 1 (52) |  |  |  |  | basal cell carcinoma (56) | 2  (71) | 70 (52-71) | 6 (52-71) | MSS | [14] | colonoscopy, gastroscopy | proctocolectomy |
| SM6  Ser478Asn | IV:8 | F |  | | 1 (45) |  |  |  |  |  |  | >30 with LGD (53-70) | 3 | - | [14] |  | total colectomy |
| SM6  Ser478Asn | V:2 | M | 1 (28) | |  |  |  |  |  | splenic fissure cancer |  | 28 (28-35) |  |  | [14] |  |  |
| SM6  Ser478Asn | V:10 | M |  | |  |  |  | 2 astro-cytomas (26) |  |  |  | 9 (33-45) | 2 (33-45) | - | [14] | colonoscopy |  |
| SM6  Ser478Asn | V:12 | M |  | |  |  |  |  |  |  |  | 17(29-46) | 50-100 hyperplastic polyps (29-46) | - | [14] | colonoscopy |  |
| SM6  Ser478Asn | VI:1 | F | unaffected age 18 | | | | | | | |  |  |  |  | New CORGI |  |  |
| SM4  Ser478Asn | III.2 | F | 2 (29 & 50) | |  |  |  |  |  |  | 1 (55) | multiple  (40-60) |  | - | [14] |  |  |
| SM4  Ser478Asn | III.6 | F |  | | 1 (50) | 1 (57) |  |  |  |  |  | >19 (41) |  | - | [14] |  |  |
| SM4  Ser478Asn | III.8 | M | 1 (32) | |  |  |  |  |  |  |  | 45 (32-52) | Multiple (32-52) |  | [14] |  | colectomy |
| SM4  Ser478Asn | III.9 | F | 1 (33) | | 1 (45) |  |  |  |  |  |  | 40 (33-49) |  | - | [14] |  | right colectomy |
| Ser478Asn S478N | 6582201 | M | 1(28) | |  |  |  |  |  |  |  | adenomas (not stated) |  |  | [9] |  |  |

POLE variants are annotated relative to transcript NM_006231.3 and protein accession NP_006222.2. POLD1 variants are annotated relative to transcript NM_001256849 and protein accession NP_001243778 Sex: M=male, F=female, U=unknown

Supplementary References:

1. Jansen, A.M., et al., *Combined mismatch repair and POLE/POLD1 defects explain unresolved suspected Lynch syndrome cancers.* Eur J Hum Genet, 2016. **24**(7): p. 1089-92.

2. Billingsley, C.C., et al., *Polymerase varepsilon (POLE) mutations in endometrial cancer: clinical outcomes and implications for Lynch syndrome testing.* Cancer, 2015. **121**(3): p. 386-94.

3. Buchanan, D.D., et al., *Risk of colorectal cancer for carriers of a germ-line mutation in POLE or POLD1.* Genet Med, 2017.

4. Shinbrot, E., et al., *Exonuclease mutations in DNA polymerase epsilon reveal replication strand specific mutation patterns and human origins of replication.* Genome Res, 2014. **24**(11): p. 1740-50.

5. Cancer Genome Atlas, N., *Comprehensive molecular characterization of human colon and rectal cancer.* Nature, 2012. **487**(7407): p. 330-7.

6. Church, D.N., et al., *DNA polymerase epsilon and delta exonuclease domain mutations in endometrial cancer.* Hum Mol Genet, 2013. **22**(14): p. 2820-8.

7. Konrad J Karczewski, L.C.F., Grace Tiao, Beryl B Cummings, Jessica Alföldi, Qingbo Wang, Ryan L Collins, Kristen M Laricchia, Andrea Ganna, Daniel P Birnbaum, Laura D Gauthier, Harrison Brand, Matthew Solomonson, Nicholas A Watts, Daniel Rhodes, Moriel Singer-Berk, Eleanor G Seaby, Jack A Kosmicki, Raymond K Walters, Katherine Tashman, Yossi Farjoun, Eric Banks, Timothy Poterba, Arcturus Wang, Cotton Seed, Nicola Whiffin, Jessica X Chong, Kaitlin E Samocha, Emma Pierce-Hoffman, Zachary Zappala, Anne H O'Donnell-Luria, Eric Vallabh Minikel, Ben Weisburd, Monkol Lek, James S Ware, Christopher Vittal, Irina M Armean, Louis Bergelson, Kristian Cibulskis, Kristen M Connolly, Miguel Covarrubias, Stacey Donnelly, Steven Ferriera, Stacey Gabriel, Jeff Gentry, Namrata Gupta, Thibault Jeandet, Diane Kaplan, Christopher Llanwarne, Ruchi Munshi, Sam Novod, Nikelle Petrillo, David Roazen, Valentin Ruano-Rubio, Andrea Saltzman, Molly Schleicher, Jose Soto, Kathleen Tibbetts, Charlotte Tolonen, Gordon Wade, Michael E Talkowski, The Genome Aggregation Database Consortium, Benjamin M Neale, Mark J Daly, Daniel G MacArthur, *Variation across 141,456 human exomes and genomes reveals the spectrum of loss-of-function intolerance across human protein-coding genes.* bioRxiv, 2019.

8. Aoude, L.G., et al., *POLE mutations in families predisposed to cutaneous melanoma.* Fam Cancer, 2015. **14**(4): p. 621-8.

9. Chubb, D., et al., *Genetic diagnosis of high-penetrance susceptibility for colorectal cancer (CRC) is achievable for a high proportion of familial CRC by exome sequencing.* J Clin Oncol, 2015. **33**(5): p. 426-32.

10. Rohlin, A., et al., *GREM1 and POLE variants in hereditary colorectal cancer syndromes.* Genes Chromosomes Cancer, 2016. **55**(1): p. 95-106.

11. Spier, I., et al., *Frequency and phenotypic spectrum of germline mutations in POLE and seven other polymerase genes in 266 patients with colorectal adenomas and carcinomas.* Int J Cancer, 2015. **137**(2): p. 320-31.

12. Elsayed, F.A., et al., *Low frequency of POLD1 and POLE exonuclease domain variants in patients with multiple colorectal polyps.* Mol Genet Genomic Med, 2019. **7**(4): p. e00603.

13. Bellido, F., et al., *POLE and POLD1 mutations in 529 kindred with familial colorectal cancer and/or polyposis: review of reported cases and recommendations for genetic testing and surveillance.* Genet Med, 2016. **18**(4): p. 325-32.

14. Palles, C., et al., *Germline mutations affecting the proofreading domains of POLE and POLD1 predispose to colorectal adenomas and carcinomas.* Nat Genet, 2013. **45**(2): p. 136-44.

15. Pelosini, C., et al., *Identification of a novel mutation in the polymerase delta 1 (POLD1) gene in a lipodystrophic patient affected by mandibular hypoplasia, deafness, progeroid features (MDPL) syndrome.* Metabolism, 2014. **63**(11): p. 1385-9.

16. Castellsague, E., et al., *Novel POLE pathogenic germline variant in a family with multiple primary tumors results in distinct mutational signatures.* Hum Mutat, 2019. **40**(1): p. 36-41.

17. Rohlin, A., et al., *A mutation in POLE predisposing to a multi-tumour phenotype.* Int J Oncol, 2014. **45**(1): p. 77-81.

18. Vande Perre, P., et al., *Germline mutation p.N363K in POLE is associated with an increased risk of colorectal cancer and giant cell glioblastoma.* Fam Cancer, 2019. **18**(2): p. 173-178.

19. Valle, L., et al., *New insights into POLE and POLD1 germline mutations in familial colorectal cancer and polyposis.* Hum Mol Genet, 2014. **23**(13): p. 3506-12.

20. Elsayed, F.A., et al., *Germline variants in POLE are associated with early onset mismatch repair deficient colorectal cancer.* Eur J Hum Genet, 2015. **23**(8): p. 1080-4.

21. Wimmer, K., et al., *A novel germline POLE mutation causes an early onset cancer prone syndrome mimicking constitutional mismatch repair deficiency.* Fam Cancer, 2017. **16**(1): p. 67-71.

22. Hansen, M.F., et al., *A novel POLE mutation associated with cancers of colon, pancreas, ovaries and small intestine.* Fam Cancer, 2015. **14**(3): p. 437-48.
